# Supplementary material for: Age-related and disease locus-specific mechanisms contribute to early remodelling of chromatin structure in Huntington’s disease mice
Source: Nat Commun. 2021 Jan 13;12:364. doi: 10.1038/s41467-020-20605-2 (PMC7807045; doi:10.1038/s41467-020-20605-2)
Supplement: Supplementary file 1 — Supplementary Information [file 41467_2020_20605_MOESM1_ESM.pdf]

Figure S1

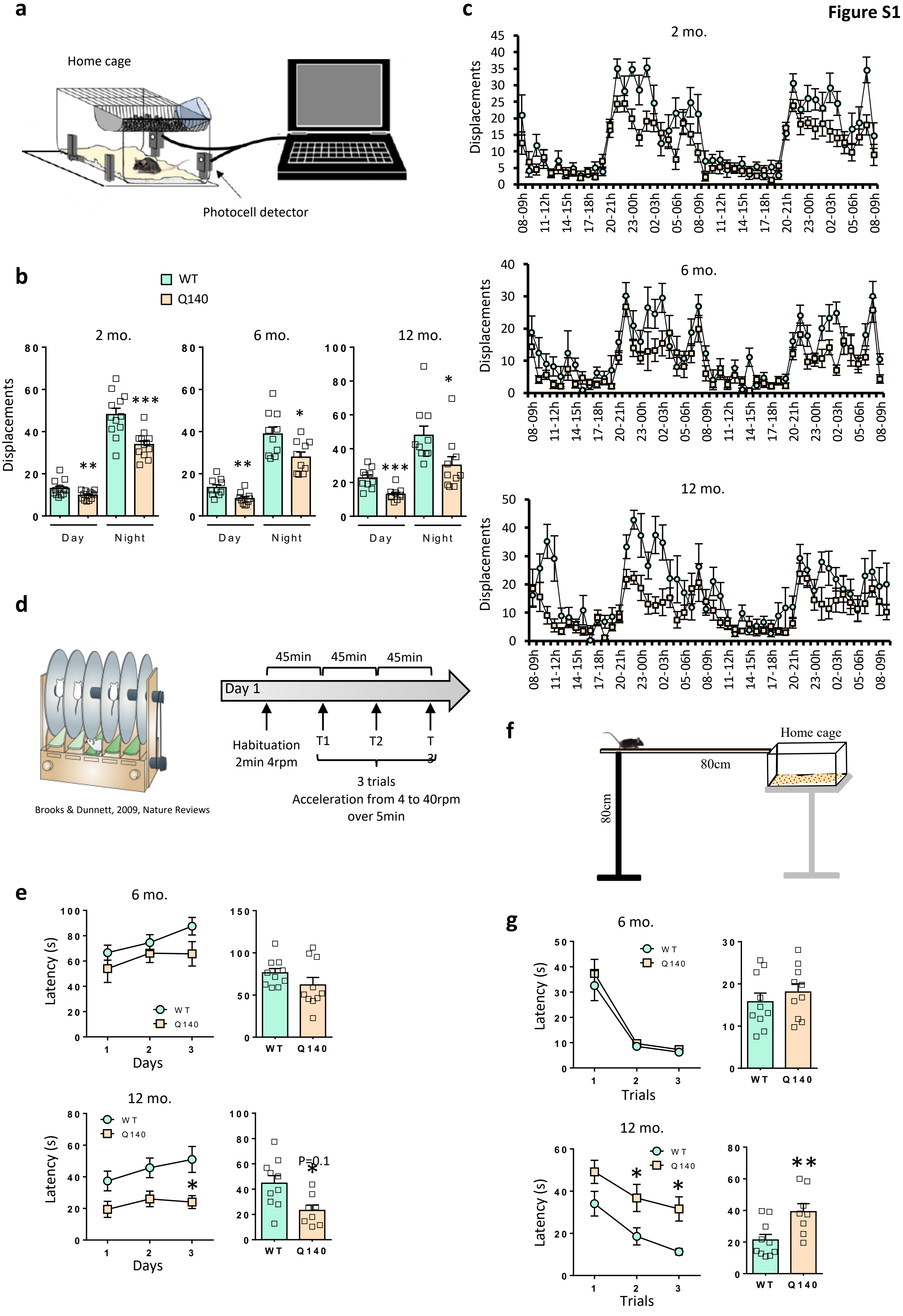
a
Home cage
Photocell detector
b
WT
Q140
2 mo.
6 mo.
12 mo.
Displacements
Day
Night
**
***
*
c
2 mo.
6 mo.
12 mo.
Displacements
08-09h
11-12h
14-15h
17-18h
20-21h
23-00h
02-03h
05-06h
d
Brooks & Dunnett, 2009, Nature Reviews
45min
45min
45min
Day 1
Habituation
2min 4rpm
T1
T2
T3
3 trials
Acceleration from 4 to 40rpm
over 5min
e
6 mo.
12 mo.
Latency (s)
Days
WT
Q140
P=0.1
f
Home cage
80cm
80cm
g
6 mo.
12 mo.
Latency (s)
Trials
WT
Q140

Supplementary Fig. 1

Behavioural analysis of HD Q140 mice

a, Scheme representing actography equipment to measure spontaneous locomotor activity. The setup is composed of two photocell detectors positioned at the front and back of the cage, connected to a computer. A longitudinal displacement is recorded when the two detectors are successively interrupted. **b**, Bar graphs showing spontaneous locomotor activity as mean ± sem of number of longitudinal displacements during the day and night phases at 2, 6 and 12 months of age for WT and Q140 het mice. n=12 WT mice and n=12 Q140 mice at 2 months; n=10 WT mice and n=10 Q140 mice at 6 months; n=10 WT and n=10 Q140 mice at 12 months. Data were analysed using unpaired two-tailed *t*-test: *$P_{2m\text{-}day}$ = 0.0167, ***$P_{2m\text{-}night}$ = 0.0005, **$P_{6m\text{-}day}$ = 0.0042, *$P_{6m\text{-}night}$ = 0.0153, ***$P_{12m\text{-}day}$ = 0.0004, *$P_{12m\text{-}night}$ = 0.028, WT vs Q140 mice. **c**, Graphs showing the mean ± sem of the number of displacements/hour for a total of 48 consecutive hours (2 days and 2 nights) at 2, 6 and 12 months of age for WT and Q140 mice. **d**, Scheme showing rotarod motor coordination test device and protocol. Mice were positioned on a rotating cylinder undergoing progressing acceleration over time (0-40 rpms over 5 min). 45 min before the test, a first habituation was performed with a constant speed of 4 rpms for 2 min and with a delay of 45 min. 3 additional trials were performed with the accelerating procedure. **e**, On the left, graphs showing the curves of tendency for latency to fall (mean ± sem) for 6 and 12 month-old WT and Q140 het mice along the 3 days of test. n=10 WT mice and n=10 Q140 mice at 6 months; n=10 WT mice and n=8 Q140 mice at 12 months. Data were analysed using one-way Anova with repeated measures; multiple comparisons used the Newman-Keuls post-hoc test, *$p < 0.05$, Q140 vs WT comparison. On the right, bar graphs showing mean latency across the 3 days of test (+/- sem). Data were analysed using unpaired two-tailed *t*-test: P_{6m} = 0.182, *P_{12m} = 0.0132, WT vs Q140 mice. **f**, Scheme showing bar test paradigm for motor coordination and balance assessment. The device is composed of a thin (1cm) raised bar (80cm above ground) that mice have to cross to reach their home cage. Latency to reach home-cage was recorded. **g**, On the left, graphs showing the latency to cross the bar (mean ± sem) over 3 different trials for WT and Q140 het mice at 6 and 12 months of age. n=10 WT mice and n=10 Q140 mice at 6 months; n=10 WT mice and n=8 Q140 mice at 12 months. Data were analysed using one-way Anova with repeated measures; multiple comparisons used the Newman-Keuls post-hoc test, *, $p<0.05$, Q140 vs WT comparison. On the right, bar graphs showing mean latency across the 3 days of test (+/- sem). Data were analysed using unpaired two-tailed *t*-test: P_{6m} = 0.4232, **P_{12m} = 0,009, WT vs Q140 mice. Source data are provided as a Source Data file.

Figure S2

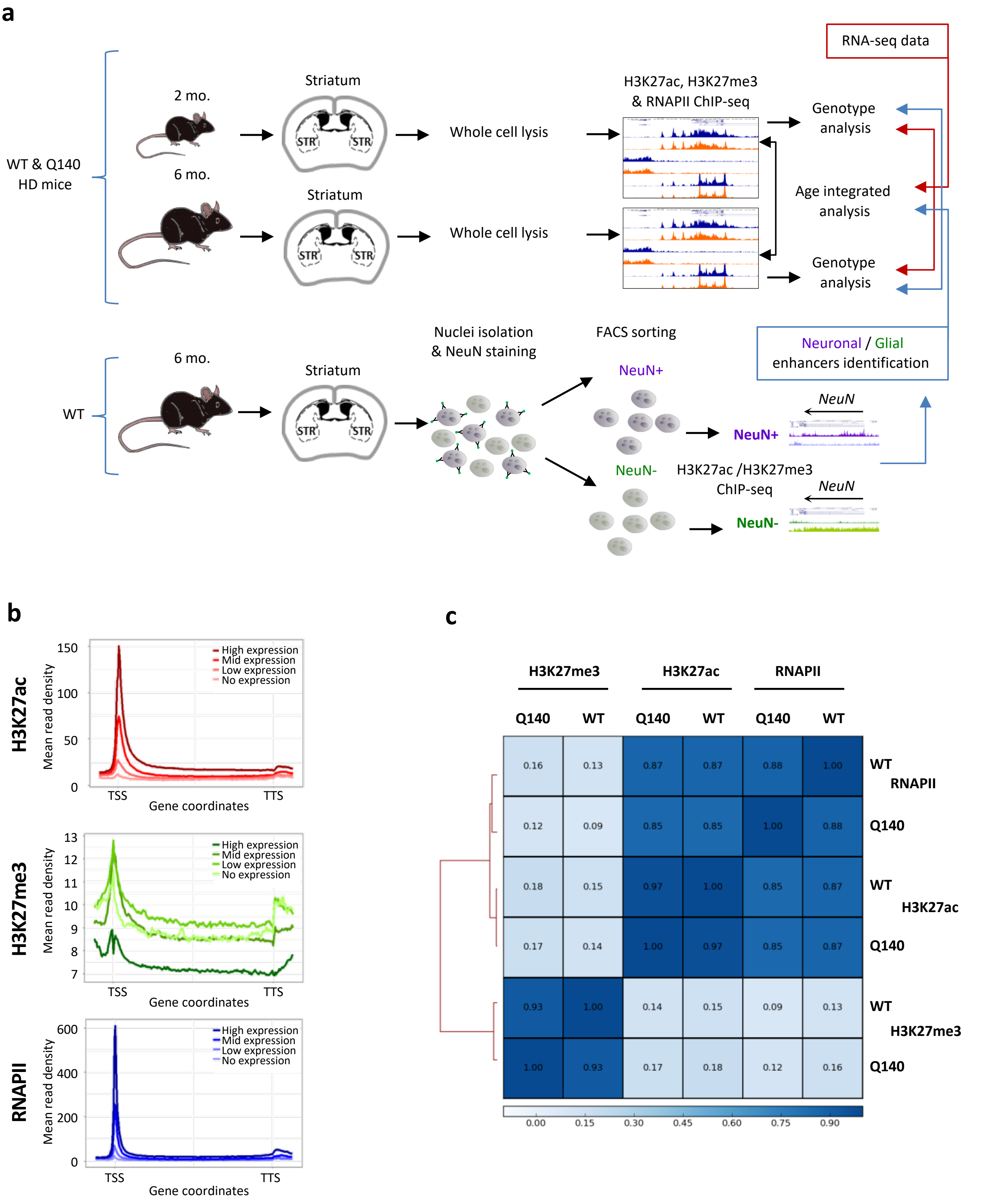

a
WT & Q140
HD mice
2 mo.
6 mo.
Striatum
STR
Whole cell lysis
H3K27ac, H3K27me3
& RNAPII ChIP-seq
RNA-seq data
Genotype
analysis
Age integrated
analysis
WT
Nuclei isolation
& NeuN staining
FACS sorting
NeuN+
NeuN-
Neuronal / Glial
enhancers identification
H3K27ac /H3K27me3
ChIP-seq
NeuN
b
H3K27ac
H3K27me3
RNAPII
Mean read density
High expression
Mid expression
Low expression
No expression
TSS
TTS
Gene coordinates
c
H3K27me3
H3K27ac
RNAPII
Q140
WT
RNAPII
H3K27ac
H3K27me3
0.16 0.13 0.87 0.87 0.88 1.00
0.12 0.09 0.85 0.85 1.00 0.88
0.18 0.15 0.97 1.00 0.85 0.87
0.17 0.14 1.00 0.97 0.85 0.87
0.93 1.00 0.14 0.15 0.09 0.13
1.00 0.93 0.17 0.18 0.12 0.16
0.00 0.15 0.30 0.45 0.60 0.75 0.90

Supplementary Fig. 2. Quality of H3K27ac, H3K27me3 and RNAPII ChIPseq data generated on bulk, neuronal and non-neuronal striatal tissue in Q140 and WT mice of 2 and 6 months: complementary information and analyses. a. Scheme showing the different ChIP-seq datasets generated using whole striatal tissue or NeuN+/NeuN- sorted nuclei from Q140 mice and integration with transcriptomic databases[5,10]. **b**, Representative gene metaprofiles for H3K27ac, H3K27me3 and RNAPII at no, low-, mid- and high-expressed genes in the striatum. No-, low-, mid- and high-expressed genes were delineated according to quartile distribution of normalized striatal RNAseq values (i.e. no-expressed < 1st, 1st <low-expressed < 2nd, 2nd<mid-expressed<3rd, high-expressed>4th quartiles). H3K27ac, H3K27me3 and RNAPII ChIPseq data and RNAseq data generated on WT striatum at 6 months were used in the analysis. TSS, Transcription Start Site; TTS, Transcription Termination Site. As expected, H3K27ac and RNAPII signals are proportional to gene expression levels, in contrast to H3K27me3. **c**, Correlation heatmap for H3K27ac, H3K27Me3 and RNAPII in WT and Q140 striatum. This example heatmap was generated using ChIPseq data generated on the striatum of WT male mice of 6 months of age, and computing read enrichment every 5Kb bins across the entire genome. Spearman correlation coefficients are provided for each regression. As expected, H3K27ac and RNAPII signals are highly correlated, in contrast to H3K27me3 with H3K27ac or RNAPII. Source data are provided as a Source Data file.

a

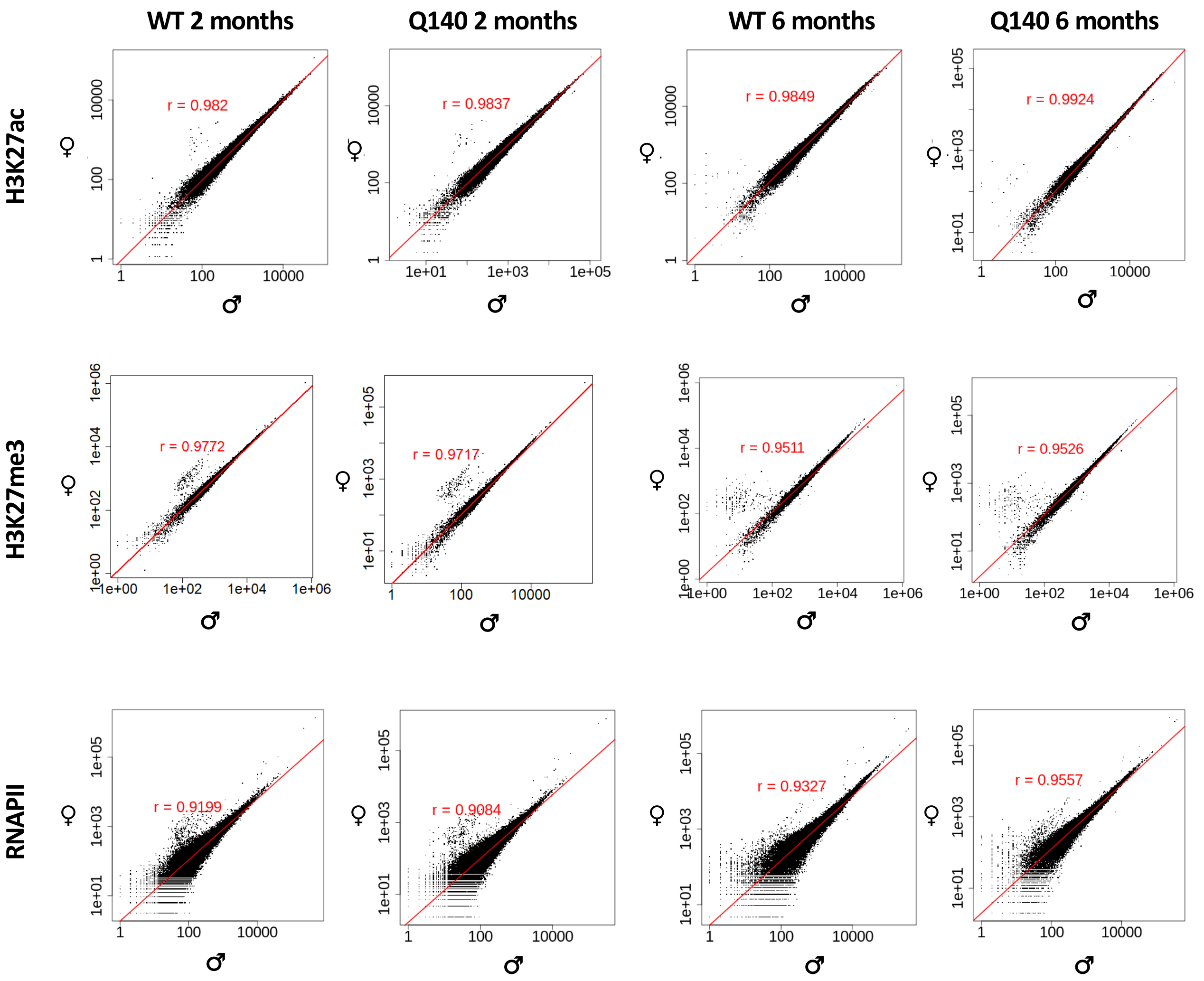
WT 2 months
Q140 2 months
WT 6 months
Q140 6 months
H3K27ac
H3K27me3
RNAPII
r = 0.982
r = 0.9837
r = 0.9849
r = 0.9924
r = 0.9772
r = 0.9717
r = 0.9511
r = 0.9526
r = 0.9199
r = 0.9084
r = 0.9327
r = 0.9557
♀
♂

b

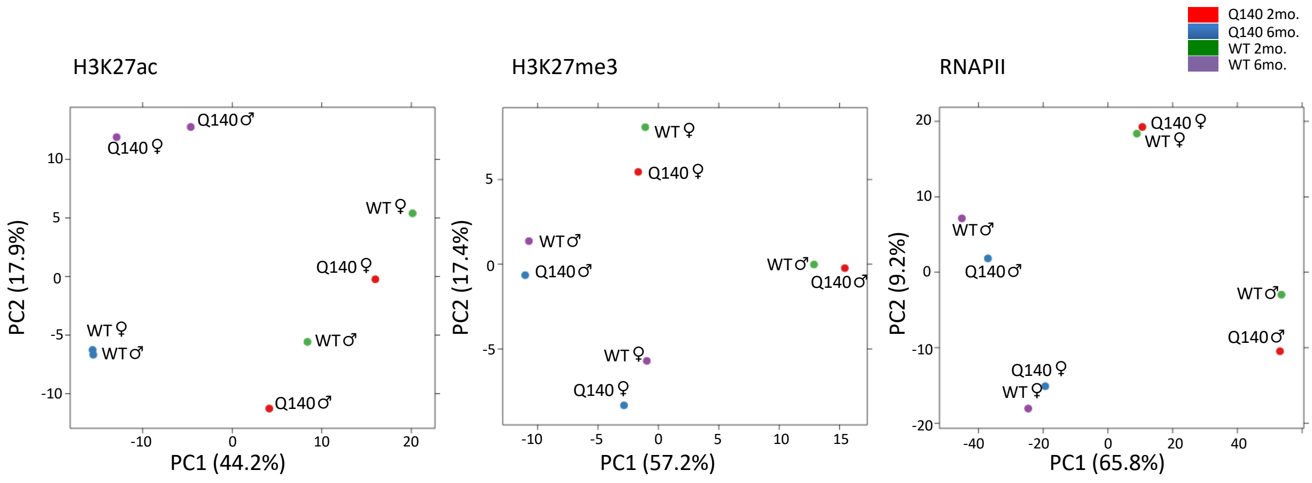
Q140 2mo.
Q140 6mo.
WT 2mo.
WT 6mo.
H3K27ac
H3K27me3
RNAPII
PC1 (44.2%)
PC2 (17.9%)
PC1 (57.2%)
PC2 (17.4%)
PC1 (65.8%)
PC2 (9.2%)
Q140♂
Q140♀
WT♀
WT♂

Supplementary Fig. 3. Exploratory analysis of of H3K27ac, H3K27me3 and RNAPII ChIPseq data generated on bulk striatal tissue in Q140 and WT mice of 2 and 6 months: complementary information and analyses

a, Scatterplots showing the comparison of ChIPseq read count per peak between male and female samples for H3K27ac (upper panel), H3K27me3 (middle panel) and RNAPII (lower panel) and for each condition (WT striatum at 2 months, WT2; Q140 striatum at 2 months, Q1402; WT striatum at 6 months, WT6; Q140 striatum at 6 months, Q1406). **b**, Variance observation between H3K27ac (left), H3K27me3 (middle) and RNAPII (right) ChIPseq datasets generated on the striatum of male and female Q140 and WT mice of 2 and 6 months. ChIPseq experiments using Q140 and WT samples of specific age and sex were performed simultaneously. Experiments performed on different sexes and at different ages were conducted at different times. Principal component analysis (PCA) was computed on variance stabilized data using the method proposed by Anders and Huber[55]. Sample colours indicate groups (Q140 2 months, red; Q140 6 months, blue; WT 2 months, green; WT 6 months, purple). First factorial plans are represented. The first axis explains the major variability between the samples, predominantly separating them according to age.

a

Q140 vs WT	Target	Down	Up
2 MONTHS	H3K27ac	305	367
	H3K27me3	35	1
	RNAPII	1,204	17
6 MONTHS	H3K27ac	2,432	3,226
	H3K27me3	193	173
	RNAPII	896	174

b

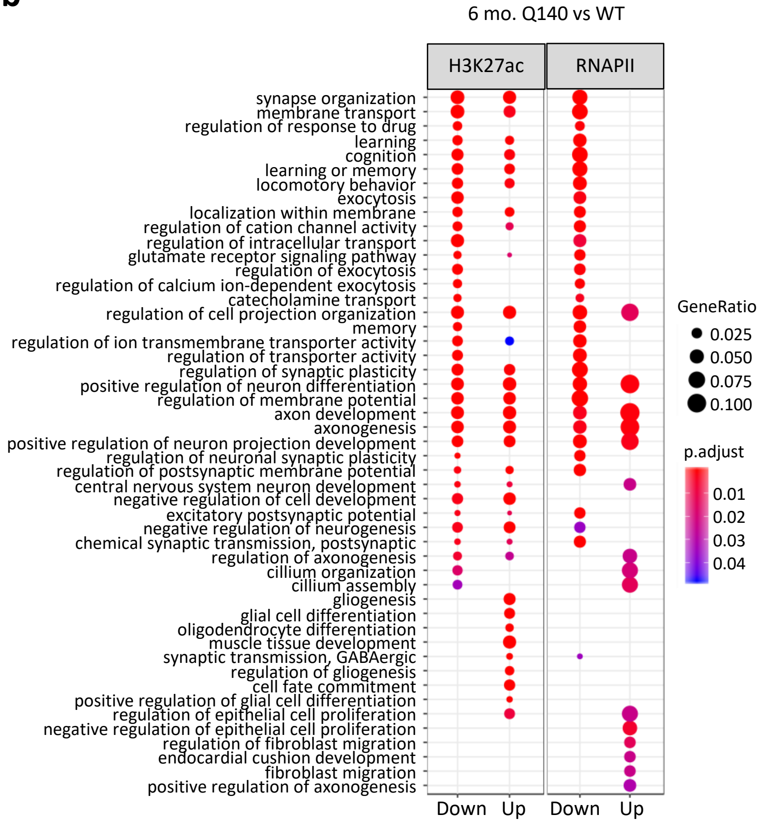

c

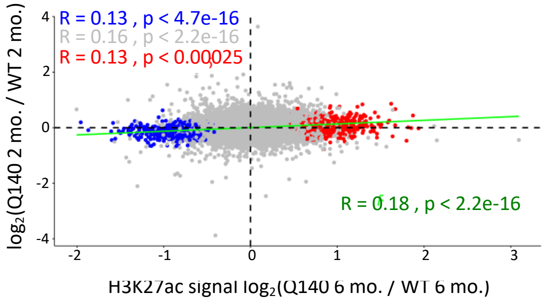

Supplementary Fig. 4

H3K27ac, H3K27me3 and RNAPII ChIPseq data generated on bulk striatal tissue in Q140 and WT mice of 2 and 6 months: complementary information and analyses

a, Summary table of regions showing differential enrichments in H3K27ac, H3K27me3 and RNAPII in Q140 vs WT striatal samples at 2 and 6 months, processing male and female samples as replicates. **b**, Gene Ontology analysis of regions differentially enriched in H3K27ac and RNAPII between Q140 and WT striatum at 6 months (FDR <0.05). Significant biological processes are shown using dot size proportional to gene ratio and heatmap reflecting adj. p value. **c**, Linear regression analysis showing the correlation between H3K27ac changes in Q140 vs WT samples at 2 and 6 months. The correlation is shown for all genes (green), as well as genes depleted (red), enriched (blue) and showing no change (grey) in H3K27ac in Q140 vs WT striatum at 6 months of age. Pearson's correlation index and p value for fitted linear model is shown. Source data are provided as a Source Data file.

a

	2 months				6 months			
	♂		♀		♂		♀	
	Down	Up	Down	Up	Down	Up	Down	Up
H3K27ac	1,478	1,939	1,193	1,874	6,610	6,643	4,567	9,962
H3K27me3	832	257	1,123	371	600	1,947	2,911	2,104
RNAPII	6,681	2,523	7,843	844	8,531	2,506	2,858	3,338

b

H3K27ac

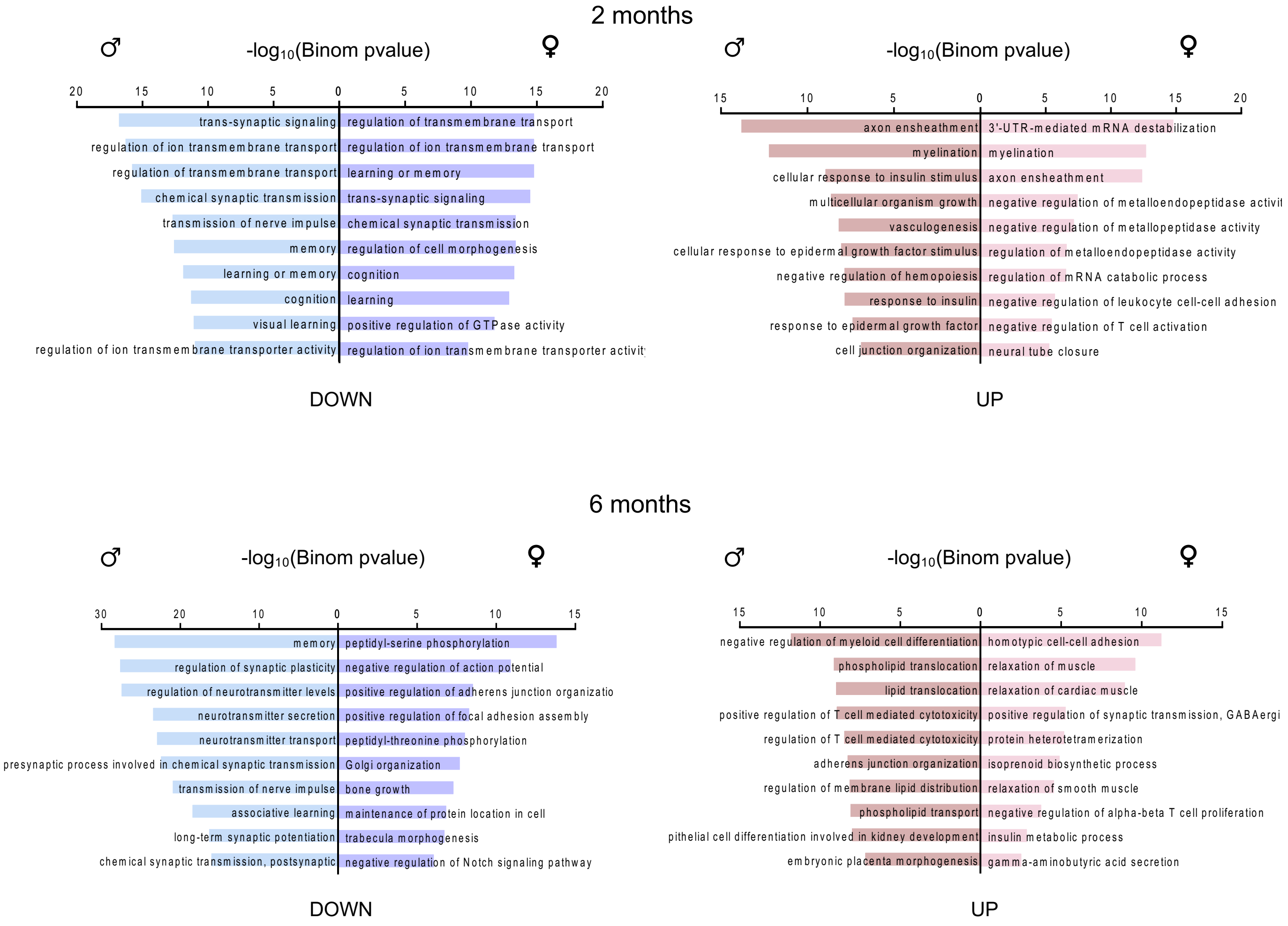

Supplementary Fig. 5

H3K27ac, H3K27me3 and RNAPII ChIPseq data generated on bulk striatal tissue in Q140 and WT mice of 2 and 6 months: independent analyses of male and female samples

a, Summary table of regions showing differential enrichments in H3K27ac, H3K27me3 and RNAPII in Q140 vs WT striatum at 2 and 6 months using separate analysis for male and female samples. **b**, Gene Ontology analysis of regions shown in summary table, differentially enriched in H3K27ac in Q140 vs WT samples (FDR <0.05). Top 10 most significant terms are shown. Source data are provided as a Source Data file.

Figure S6

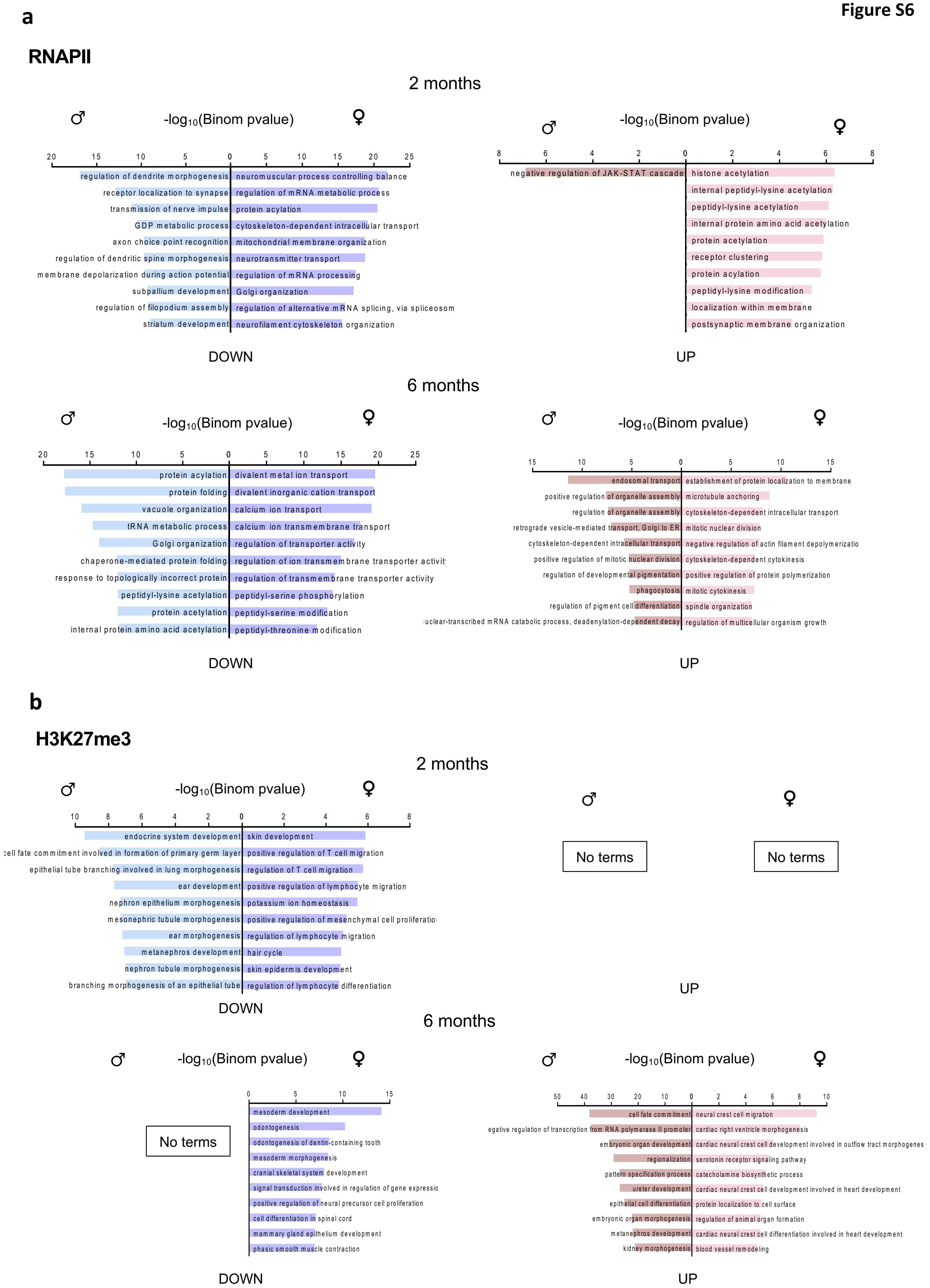
a
RNAPII
2 months
♂ -log10(Binom pvalue) ♀
regulation of dendrite morphogenesis
receptor localization to synapse
transmission of nerve impulse
GDP metabolic process
axon choice point recognition
regulation of dendritic spine morphogenesis
membrane depolarization during action potential
subpallium development
regulation of filopodium assembly
striatum development
neuromuscular process controlling balance
regulation of mRNA metabolic process
protein acylation
cytoskeleton-dependent intracellular transport
mitochondrial membrane organization
neurotransmitter transport
regulation of mRNA processing
Golgi organization
regulation of alternative mRNA splicing, via spliceosom
neurofilament cytoskeleton organization
DOWN
negative regulation of JAK-STAT cascade
histone acetylation
internal peptidyl-lysine acetylation
peptidyl-lysine acetylation
internal protein amino acid acetylation
protein acetylation
receptor clustering
protein acylation
peptidyl-lysine modification
localization within membrane
postsynaptic membrane organization
UP
6 months
protein acylation
protein folding
vacuole organization
tRNA metabolic process
Golgi organization
chaperone-mediated protein folding
response to topologically incorrect protein
peptidyl-lysine acetylation
protein acetylation
internal protein amino acid acetylation
divalent metal ion transport
divalent inorganic cation transport
calcium ion transport
calcium ion transmembrane transport
regulation of transporter activity
regulation of ion transmembrane transporter activit
regulation of transmembrane transporter activity
peptidyl-serine phosphorylation
peptidyl-serine modification
peptidyl-threonine modification
DOWN
endosomal transport
positive regulation of organelle assembly
regulation of organelle assembly
retrograde vesicle-mediated transport, Golgi to ER
cytoskeleton-dependent intracellular transport
positive regulation of mitotic nuclear division
regulation of developmental pigmentation
phagocytosis
regulation of pigment cell differentiation
uclear-transcribed mRNA catabolic process, deadenylation-dependent decay
establishment of protein localization to membrane
microtubule anchoring
cytoskeleton-dependent intracellular transport
mitotic nuclear division
negative regulation of actin filament depolymerizatio
cytoskeletal-dependent cytokinesis
positive regulation of protein polymerization
mitotic cytokinesis
spindle organization
regulation of multicellular organism growth
UP
b
H3K27me3
2 months
endocrine system development
cell fate commitment involved in formation of primary germ layer
epithelial tube branching involved in lung morphogenesis
ear development
nephron epithelium morphogenesis
mesonephric tubule morphogenesis
ear morphogenesis
metanephros development
nephron tubule morphogenesis
branching morphogenesis of an epithelial tube
skin development
positive regulation of T cell migration
regulation of T cell migration
positive regulation of lymphocyte migration
potassium ion homeostasis
positive regulation of mesenchymal cell proliferatio
regulation of lymphocyte migration
hair cycle
skin epidermis development
regulation of lymphocyte differentiation
DOWN
No terms
No terms
UP
6 months
No terms
mesoderm development
odontogenesis
odontogenesis of dentin-containing tooth
mesoderm morphogenesis
cranial skeletal system development
signal transduction involved in regulation of gene expressio
positive regulation of neural precursor cell proliferation
cell differentiation in spinal cord
mammary gland epithelium development
phasic smooth muscle contraction
DOWN
cell fate commitment
egative regulation of transcription from RNA polymerase II promoter
embryonic organ development
regionalization
pattern specification process
ureter development
epithelial cell differentiation
embryonic organ morphogenesis
metanephros development
kidney morphogenesis
neural crest cell migration
cardiac right ventricle morphogenesis
cardiac neural crest cell development involved in outflow tract morphogenes
serotonin receptor signaling pathway
catecholamine biosynthetic process
cardiac neural crest cell development involved in heart development
protein localization to cell surface
regulation of animal organ formation
cardiac neural crest cell differentiation involved in heart development
blood vessel remodeling
UP

Supplementary Fig. 6

RNAPII and H3K27me3 ChIPseq data generated on bulk striatal tissue in Q140 and WT mice of 2 and 6 months: independent analyses of male and female samples

a, **b**, Gene Ontology analysis of regions shown in summary table presented in supplementary Fig. 5a, differentially enriched in RNAPII (a) and H3K27me3 (b) in Q140 vs WT samples (FDR <0.05). Top 10 most significant terms are shown. Source data are provided as a Source Data file.

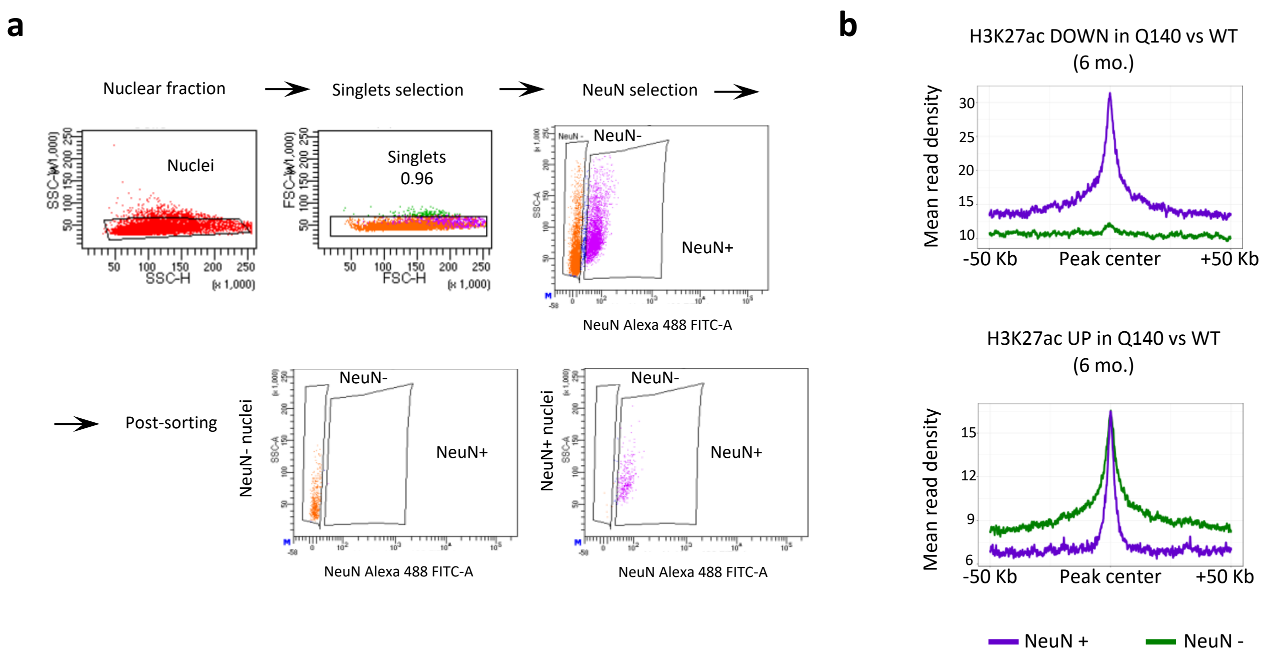

Supplementary Fig. 7

H3K27ac, H3K27me3 and RNAPII ChIPseq data generated on neuronal and non-neuronal striatal tissue in Q140 and WT mice of 2 and 6 months: complementary information and analyses

a, Representative data showing flow cytometry results for sorting of striatal nuclei stained with NeuN neuronal marker. An initial gating using side scatter (SSC) of height (SSC-H) versus width (SSC-W) of the pulse signal detected was used to separate events corresponding to nuclear populations. A second gating strategy using forward scatter (FSC) of height (FSC-H) versus width (FSC-W) of the pulse signal detected was used to separate singlet events from doublet or multiplet nuclei. For NeuN +/- nuclear separation, NeuN channel signal (Alexa 488 FITC-A or Alexa 405) versus side scatter area (SSC-A) gating strategy was used. Post-sorting analysis of purified NeuN+ and NeuN- populations is shown as a control for purity assessment of sorted populations. **b**, Metaprofiles showing H3K27ac read count distribution in NeuN+ and NeuN- sorted ChIP-seq data, using differentially enriched peaks in Q140 vs WT striatum at 6 months. Source data are provided as a Source Data file.

Figure S8

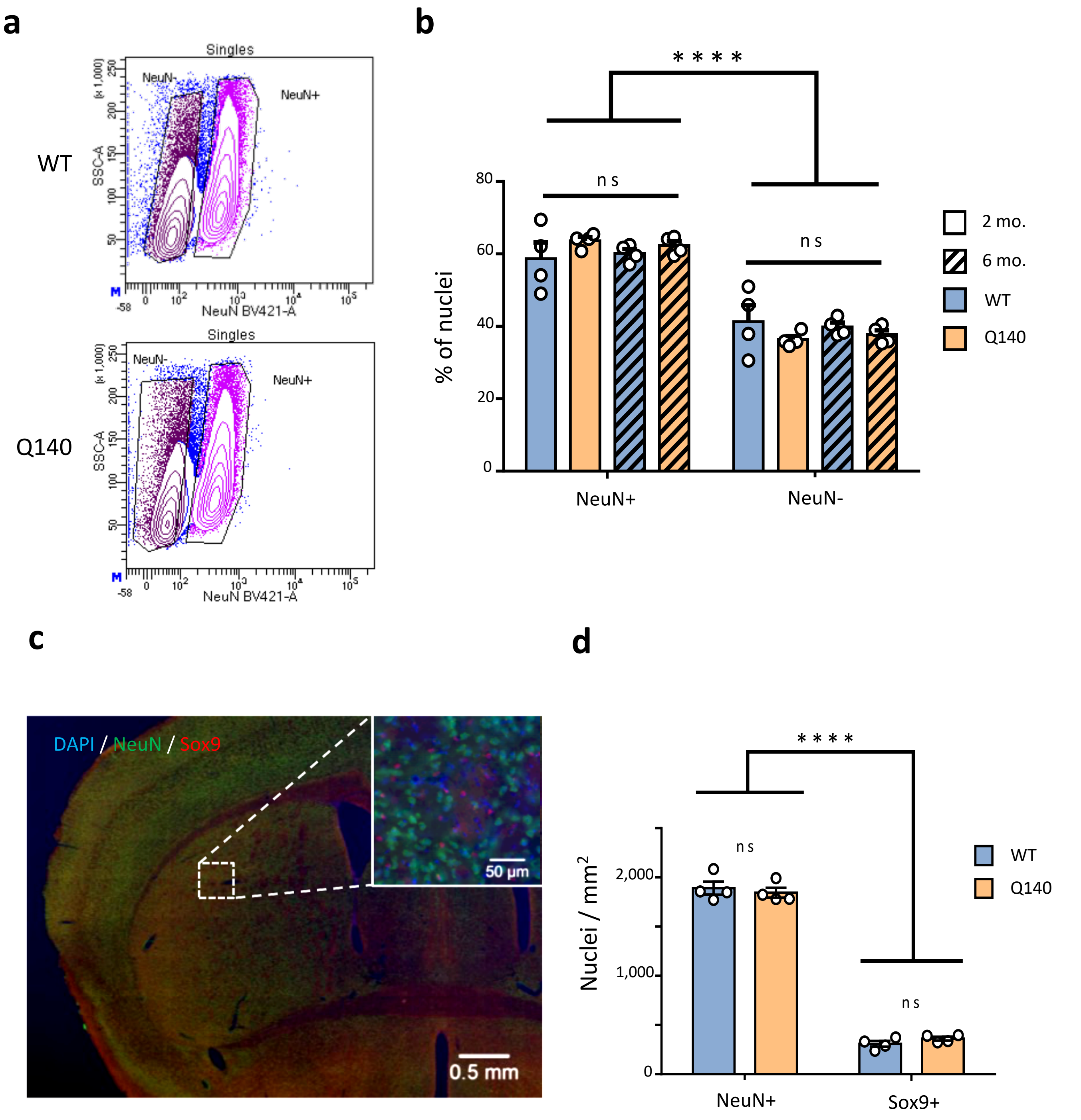
a
WT
Q140
Singles
NeuN-
NeuN+
SSC-A
(x 1,000)
NeuN BV421-A
b
% of nuclei
NeuN+
NeuN-
n s
* * * *
2 mo.
6 mo.
WT
Q140
c
DAPI / NeuN / Sox9
50 µm
0.5 mm
d
Nuclei / mm2
NeuN+
Sox9+
n s
* * * *
WT
Q140

Supplementary Fig. 8

The striatum of Q140 mice does not display significant alteration of the proportion of neuronal and glial cells at early disease stages

a-b, Neuronal ($NeuN^+$) and non-neuronal (NeuN-) nuclear populations in the striatum of WT and Q140 mice at 2 and 6 months were analysed using the FANS approach. **a**, Representative dotplot showing the distribution of $NeuN^+$ (pink, right side) and NeuN- (purple, left side) singlet events in WT (top) and Q140 (bottom) striatum. **b**, Bargraphs showing the relative percentage of NeuN+ vs NeuN- nuclei as a mean ± sem in WT (blue) and Q140 (orange) striatum at 2 (clear background) and 6 (diagonal stripes) months of age (n=4 animals per group). Individual values are depicted as white circles. Data were analysed using two-way Anova; multiple comparisons used the Tukey's post-hoc test, ****, $p<0.0001$, $NeuN^+$ vs $NeuN^-$ comparison. **c-d**, Immunohistological analysis of NeuN (neuronal marker) and Sox9 (astrocytic marker) in WT and Q140 striatum at 2 months of age (n= 4 animals per group). **c**, Representative image showing the staining pattern of NeuN (green), Sox9 (red) and DAPI (blue) in WT striatum. In the upper right corner, a 40x magnification is shown for better visualization of the absence of co-localization between $NeuN^+$ and $Sox9^+$ nuclei. **e**, Bargraphs showing the number of nuclei per mm^2 as a mean ± sem of $NeuN^+$ and $Sox9^+$ populations in WT (blue) and Q140 (orange) striatum at 2 months of age. Individual values are depicted as white circles. Data were analysed using two-way Anova; multiple comparisons used the Tukey's post-hoc test, ****, $p<0.0001$, $NeuN^+$ vs $Sox9^+$ comparison. Source data are provided as a Source Data file.

Figure S9

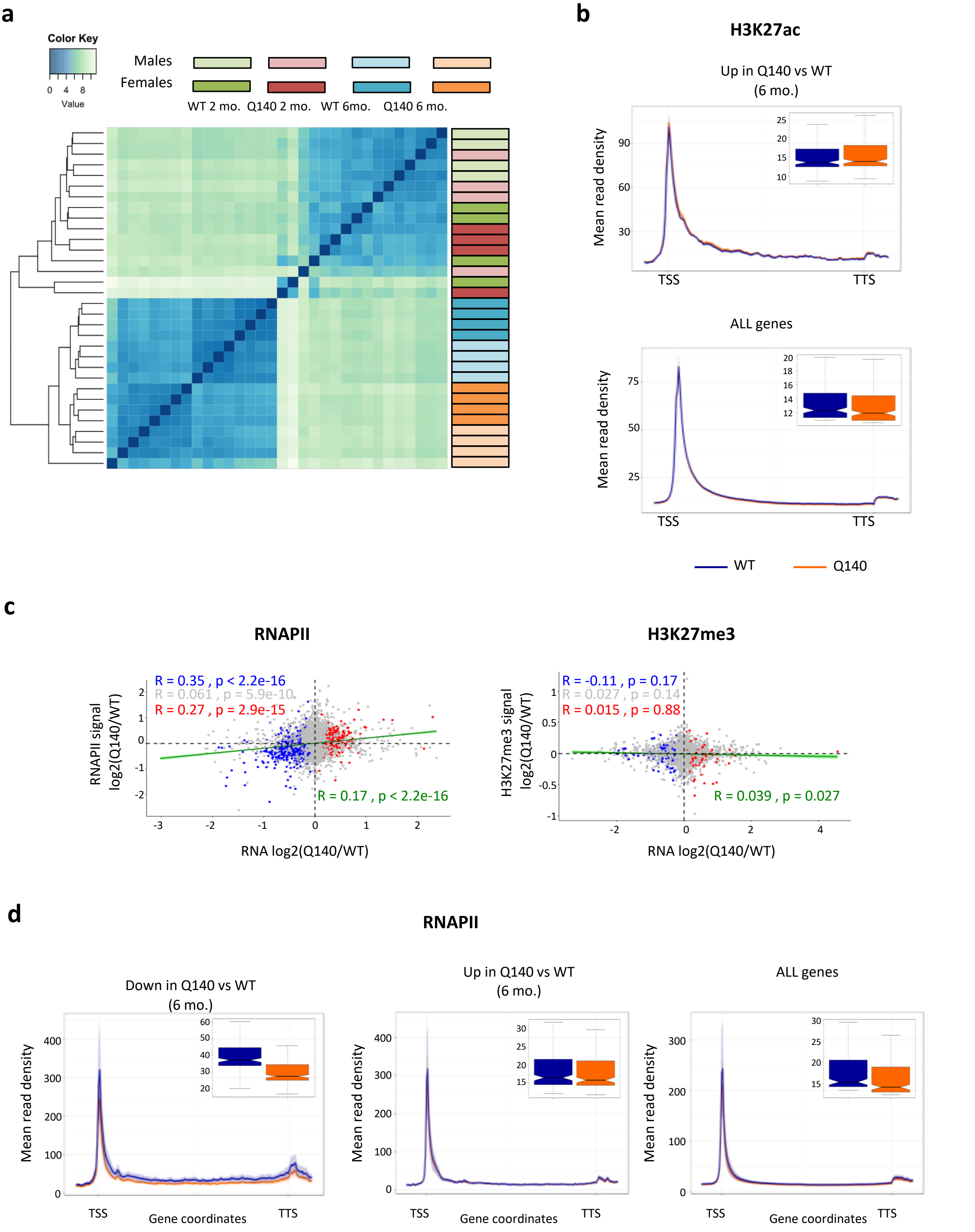
a
Color Key
0 4 8
Value
Males
Females
WT 2 mo.
Q140 2 mo.
WT 6mo.
Q140 6 mo.
b
H3K27ac
Up in Q140 vs WT
(6 mo.)
Mean read density
90
60
30
TSS
TTS
25
20
15
10
ALL genes
Mean read density
75
50
25
TSS
TTS
20
18
16
14
12
WT
Q140
c
RNAPII
R = 0.35 , p < 2.2e-16
R = 0.061 , p = 5.9e-10
R = 0.27 , p = 2.9e-15
R = 0.17 , p < 2.2e-16
RNAPII signal
log2(Q140/WT)
RNA log2(Q140/WT)
H3K27me3
R = -0.11 , p = 0.17
R = 0.027 , p = 0.14
R = 0.015 , p = 0.88
R = 0.039 , p = 0.027
H3K27me3 signal
log2(Q140/WT)
RNA log2(Q140/WT)
d
RNAPII
Down in Q140 vs WT
(6 mo.)
Up in Q140 vs WT
(6 mo.)
ALL genes
Mean read density
TSS
Gene coordinates
TTS

Supplementary Fig. 9

The HD mutation early affects epigenetic and transcriptional regulations in mouse striatum: complementary information and analyses

a, Heatmap showing hierarchical clustering of RNAseq striatal samples from Q140 mice at 2 and 6 months of age [5]. The heatmap was computed using UPGMA algorithm (Unweighted Pair Group Method with Arithmetic Mean) with the SERE coefficient as distance measure [54]. **b**, Gene body metaprofiles representing H3K27ac read count distribution for top 300 up-regulated genes in Q140 mouse striatum at 6 months and considering all genes (n= 36,873 refseq transcripts). TSS, Transcription Start Site; TTS, Transcription Termination Site. Data from male and female samples were used to generate average profile. Boxplots represent the distribution of mean read density along the profiles and show median, first quartile (Q1), third quartile (Q3) and range (min, Q1-1.5x(Q3-Q1); max, Q3+1.5x(Q3-Q1)). **c,** Linear regression analysis between transcriptional and RNAPII changes (left) and between transcriptional and H3K27me3 changes (right) in the striatum of Q140 vs WT mice of 6 months. The correlation is shown for all genes (green), genes significantly down-regulated (Fold change (FC)<1 and adj. p value <0.05; blue), genes significantly up-regulated (FC>1 and adj. p value <0.05; red) and non-significantly altered genes (grey). Pearson's correlation index and p value for fitted linear model are shown. **d**, Gene body metaprofiles representing RNAPII read count distribution for top 300 down-regulated, 300 up-regulated genes in Q140 mouse striatum at 6 months and considering all genes (n=36,873 refseq transcripts). TSS, Transcription Start Site; TTS, Transcription Termination Site. Data from male and female samples were used to generate average profile. Boxplots represent the distribution of mean read density along the profiles and show median, first quartile (Q1), third quartile (Q3) and range (min, Q1-1.5x(Q3-Q1); max, Q3+1.5x(Q3-Q1)). Source data are provided as a Source Data file.

Figure S10

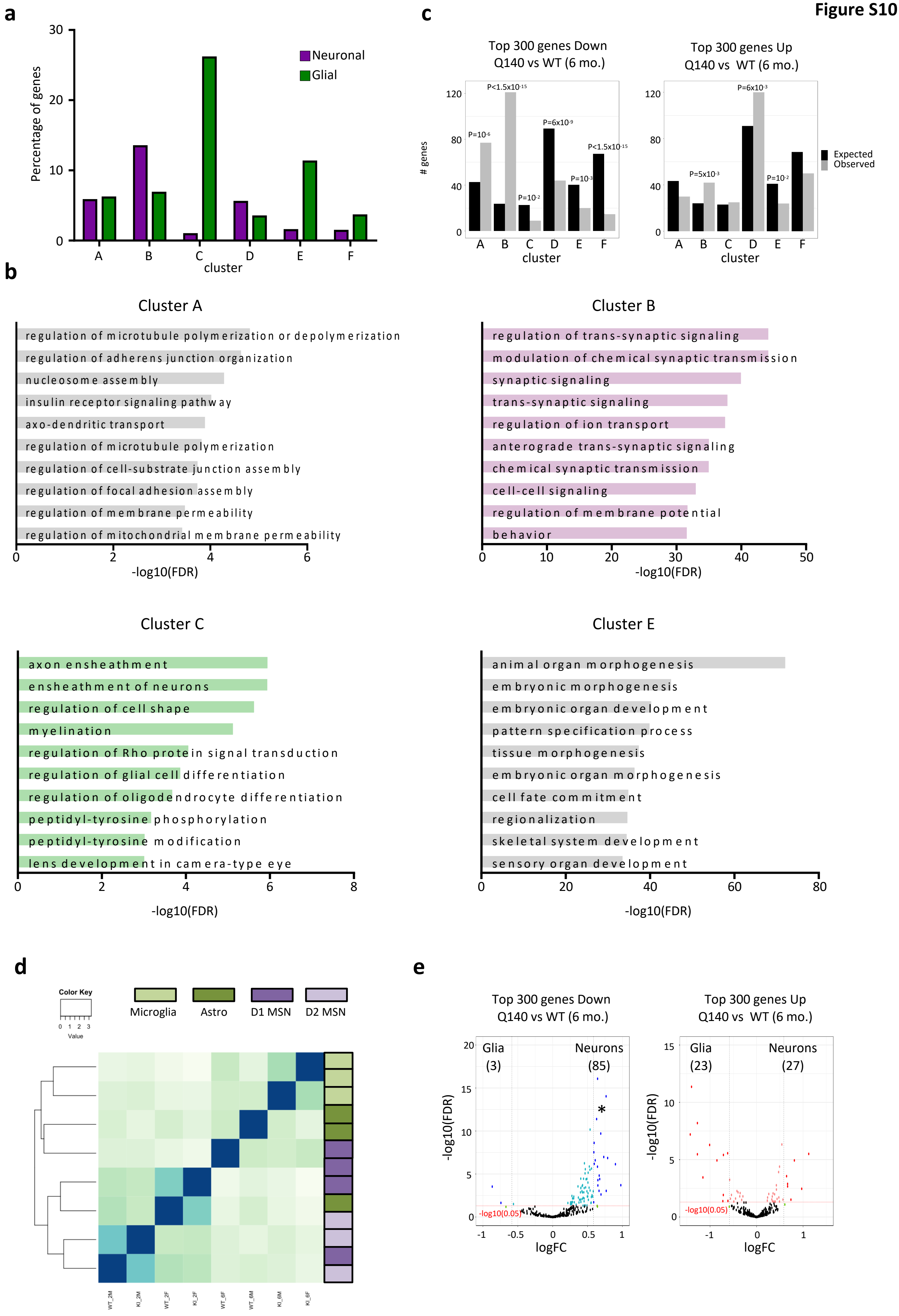

a
Percentage of genes
Neuronal
Glial
cluster
A B C D E F
c
Top 300 genes Down
Q140 vs WT (6 mo.)
Top 300 genes Up
Q140 vs WT (6 mo.)
# genes
P=10-6
P<1.5x10-15
P=10-2
P=6x10-9
P=10-3
P<1.5x10-15
P=5x10-3
P=6x10-3
P=10-2
Expected
Observed
cluster
b
Cluster A
regulation of microtubule polymerization or depolymerization
regulation of adherens junction organization
nucleosome assembly
insulin receptor signaling pathway
axo-dendritic transport
regulation of microtubule polymerization
regulation of cell-substrate junction assembly
regulation of focal adhesion assembly
regulation of membrane permeability
regulation of mitochondrial membrane permeability
-log10(FDR)
Cluster B
regulation of trans-synaptic signaling
modulation of chemical synaptic transmission
synaptic signaling
trans-synaptic signaling
regulation of ion transport
anterograde trans-synaptic signaling
chemical synaptic transmission
cell-cell signaling
regulation of membrane potential
behavior
-log10(FDR)
Cluster C
axon ensheathment
ensheathment of neurons
regulation of cell shape
myelination
regulation of Rho protein signal transduction
regulation of glial cell differentiation
regulation of oligodendrocyte differentiation
peptidyl-tyrosine phosphorylation
peptidyl-tyrosine modification
lens development in camera-type eye
-log10(FDR)
Cluster E
animal organ morphogenesis
embryonic morphogenesis
embryonic organ development
pattern specification process
tissue morphogenesis
embryonic organ morphogenesis
cell fate commitment
regionalization
skeletal system development
sensory organ development
-log10(FDR)
d
Color Key
Value
Microglia
Astro
D1 MSN
D2 MSN
WT_2M
KI_2M
WT_2F
KI_2F
WT_6F
WT_6M
KI_6M
KI_6F
e
Top 300 genes Down
Q140 vs WT (6 mo.)
Top 300 genes Up
Q140 vs WT (6 mo.)
Glia
(3)
Neurons
(85)
Glia
(23)
Neurons
(27)
-log10(FDR)
-log10(0.05)
logFC

Supplementary Fig. 10

The HD mutation early affects epigenetic and transcriptional regulations of neuronal- and glial-specific genes in mouse striatum: complementary information and analyses

a, Histograms showing the percentage of neuronal-specific genes (purple) and glial-specific genes (green) in each cluster, as generated in Fig. 2c. As expected, cluster B (neuronal super enhancers) is enriched in neuronal-specific genes and cluster C (glial super enhancers) is enriched in glial-specific genes. **b**, Bar graphs showing enriched GO terms (Biological Process, $-\log_{10}$(FDR)<0.05) in cluster A, cluster B, cluster C and cluster E. **c**, Histograms showing cluster distribution of genes down- (upper panel) and up-regulated (lower panel) in Q140 vs WT striatum at 6 months of age. 300 top dysregulated genes were analysed from RNAseq data [5]. Observed numbers were compared with expected numbers and a binomial test was used to assess significant differences, with multiple testing correction using the Bonferroni method. **d**, Heatmap showing hierarchical clustering of RNAseq samples generated using different neuronal and glial cell populations of WT mouse striatum, including medium spiny neurons expressing dopamine D1 receptor (D1 MSN), medium spiny neurons expressing dopamine D2 receptor (D2 MSN), astrocytes (Astro) and microglia (Micro) [8]. The heatmap was computed using UPGMA algorithm (Unweighted Pair Group Method with Arithmetic Mean) with the SERE coefficient as distance measure **e**, Volcano plot representation of differential expression values between glial cells (astrocytes and microglia) and neurons (Medium Spiny Neurons, MSN, including D1 and D2 MSN) using genes down (left) and up (right) in Q140 vs WT striatum at 6 months. Genes down (upper panel)/up (lower panel) in Q140 vs WT striatum and significantly increased in neurons vs glial cells ($FC>1$ and adj. p value <0.05) are shown in blue; genes down (upper panel)/up (lower panel) in Q140 vs WT striatum and significantly decreased in neurons vs glial cells ($FC<1$ and adj. p value <0.05) are shown in red. A binomial test (two-sided) was performed to assessed enrichment in neurons or glia; * $P < 2x10^{-16}$. Adjustment for multiple comparisons was not performed. RNAseq data were from [5,10]. Source data are provided as a Source Data file.

Figure S11

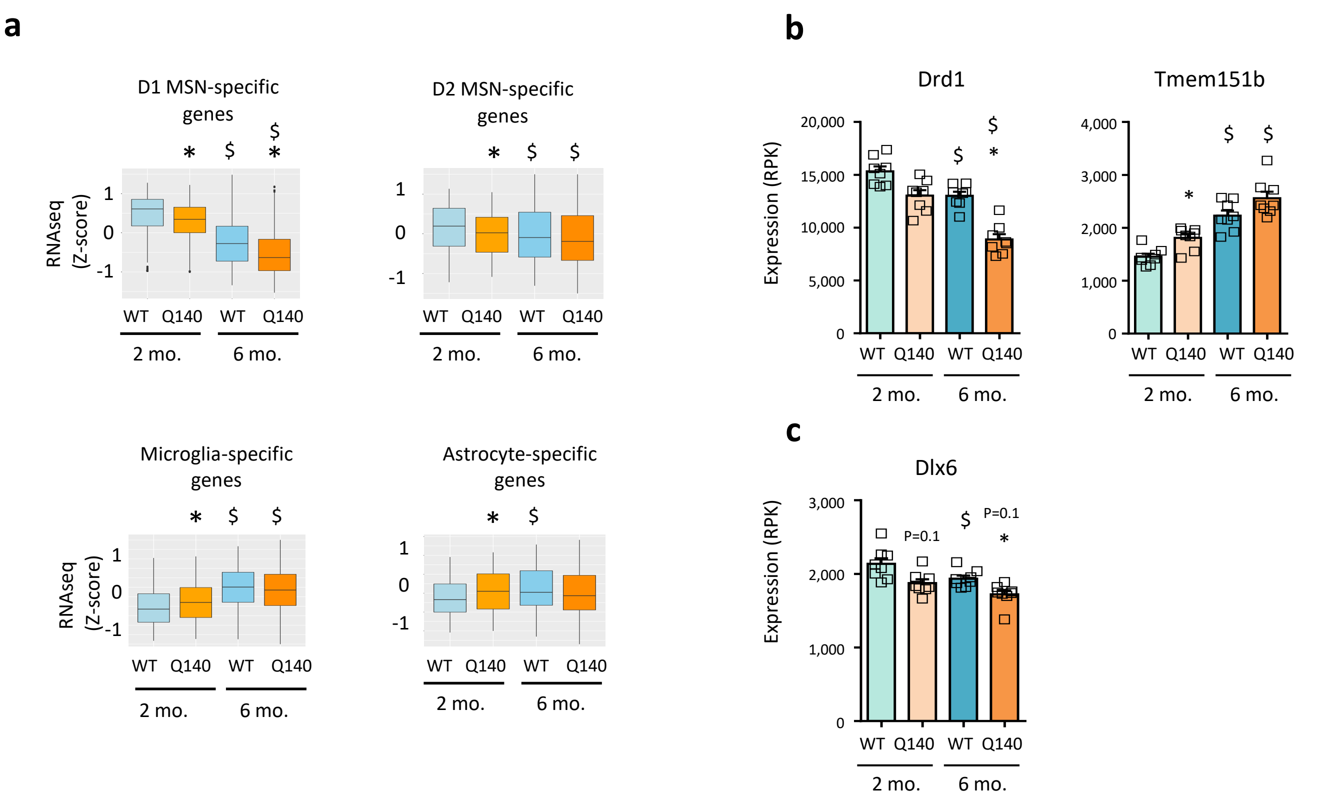

Supplementary Fig. 11

Effect of the HD mutation on age-related transcriptional changes at cell type-specific striatal genes: complementary information and analyses

a, Boxplots representing z-score values computed from RNAseq data generated on Q140 and WT striatum at 2 months and 6 months for genes increased in D1 MSN (D1-specific genes; top left), genes increased in D2 MSN (D2-specific genes; top right), genes increased in microglia (microglia-specific genes; bottom left), genes increased in astrocytes (astro-specific genes; bottom middle) and considering all genes (bottom right). Boxplots show median, first quartile (Q1), third quartile (Q3) and range (min, Q1-1.5x(Q3-Q1); max, Q3+1.5x(Q3-Q1)). Statistical analysis was performed using Kruskal-Wallis test (one-sided), with multiple testing correction using the Benjamini-Hochberg method. D1 MSN-specific genes: *, $P<2\times10^{-16}$, Q140 vs WT comparison at 2 months; *, $P=10^{-12}$, Q140 vs WT comparison at 6 months; $, $P< 2\times10^{-16}$, 6 vs 2 month comparison in WT; $, $P< 2\times10^{-16}$, 6 vs 2 month comparison in R6/1. D2 MSN-specific genes: *, $P=2\times10^{-8}$, Q140 vs WT comparison at 2 months; $, $P=3\times10^{-5}$, 6 vs 2 month comparison in WT; $, $P=2\times10^{-2}$, 6 vs 2 month comparison in R6/1. Microglia-specific genes: *, $P=3\times10^{-7}$, Q140 vs WT comparison at 2 months; $, $P< 2\times10^{-16}$, 6 vs 2 month comparison in WT; $, $P< 2\times10^{-16}$, 6 vs 2 month comparison in R6/1. Astrocyte-specific genes: *, $P=2\times10^{-5}$, Q140 vs WT comparison at 2 months; $, $P= 9\times10^{-7}$, 6 vs 2 month comparison in WT. RNAseq data from transcriptomic databases[5,10] were used for these analyses. **b,c,** Bar graphs showing mean (+/- sem) expression of *Drd1*, *Tmem151b* (c), and *Dlx6* (d) in WT and Q140 striatum at 2 and 6 months of age. Expression values were computed from re-analysis of RNAseq data from Q140 trancriptomic database [5], using DESeq2 Bioconductor package. N=8 biological replicates per group. RPK, reads per kilobases. The Benjamini and Hochberg method was used for multiple testing correction. Drd1: *, adj. p val = 5×10^{-10} Q140 vs WT at 6 months; $, adj. p val = 10^{-2} 6 vs 2 months in WT; $, adj. p val= 3×10^{-11} 6 vs 2 months in Q140. Tmem151b: *, adj. p val = 3×10^{-2} Q140 vs WT at 2 months; $, adj. p val = 3×10^{-12} 6 vs 2 months in WT; $, adj. p val= 2×10^{-8} 6 vs 2 months in Q140. Dlx6: adj. p val = 10^{-1} Q140 vs WT at 2 months; *, adj. p val = 6×10^{-3} Q140 vs WT at 6 months; $, adj. p val = 5×10^{-2} 6 vs 2 months in WT; adj. p val $=10^{-1}$ 6 vs 2 months in Q140. Source data are provided as a Source Data file.

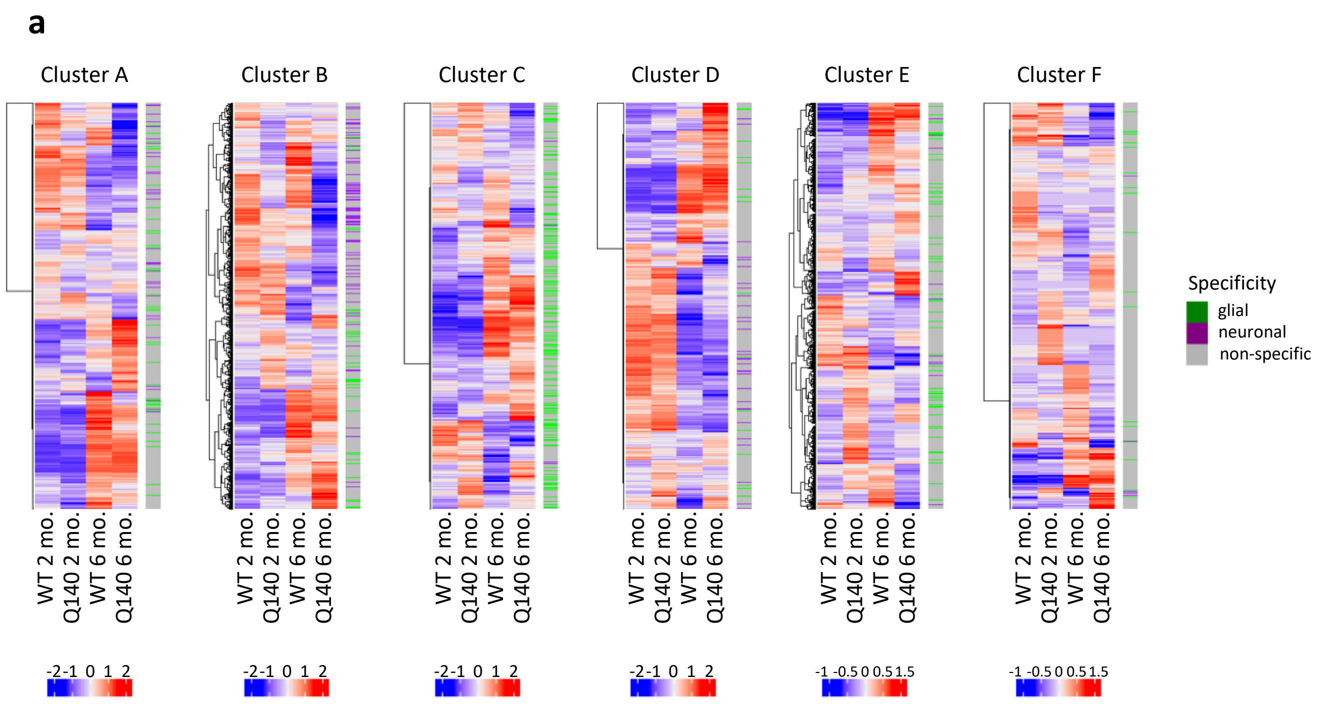

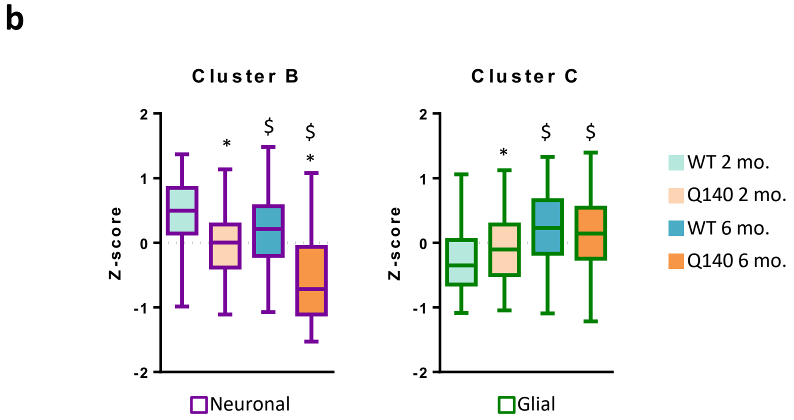

Supplementary Fig. 12

Effect of the HD mutation on age-related transcriptional changes at epigenetically defined striatal identity genes: complementary information and analyses

a, Heatmap showing hierarchical clustering of expression values in WT and Q140 striatum at 2 and 6 months of genes within the different clusters defined in Fig. 2c, using cell type-specific H3K27ac and H3K27me3 ChIPseq data. Expression values correspond to mean z-scores and were computed from RNAseq data from transcriptomic database[5]. Additionally, genes in each cluster were intersected with cell-type specific striatal RNAseq data [10]. **b**, Boxplots representing z-score values computed from RNAseq data generated on Q140 and WT striatum at 2 months and 6 months, using genes in cluster B (neuronal identity genes, left; N= 235 genes) and genes in cluster C (glial identity genes, right; N= 432 genes). Boxplots show median, first quartile (Q1), third quartile (Q3) and range (min, Q1-1.5x(Q3-Q1); max, Q3+1.5x(Q3-Q1)). Statistical analysis was performed using Kruskal-Wallis test (one-sided), with multiple testing correction using the Dunn's test. *, $P<2x10^{-4}$, Q140 vs WT comparison; $, $P<2x10^{-4}$, 6 vs 2 month comparison. RNAseq data from transcriptomic databases[5,10] were used for these analyses. The results show that down-regulation with age of neuronal identity genes in cluster B is accelerated in Q140 striatum when compared to WT striatum, whereas up-regulation of glial identity genes in cluster C with age is accelerated in Q140 striatum. Source data are provided as a Source Data file.

a

Age-associated changes in WT and Q140

PEAKS	WT		Q140	
	DOWN	UP	DOWN	UP
H3K27ac	1,126	1,19 0	2,316	1,21 8
RNAPII	5,056	3,17 4	3,975	1,52 4
H3K27me3	468	88	800	181

GENES (protein coding)	WT		Q140	
	DOWN	UP	DOWN	UP
H3K27ac	568	1,10 7	1,239	1,04 2
RNAPII	1,603	2,89 5	1,298	1,39 8
H3K27me3	367	72	609	122

b

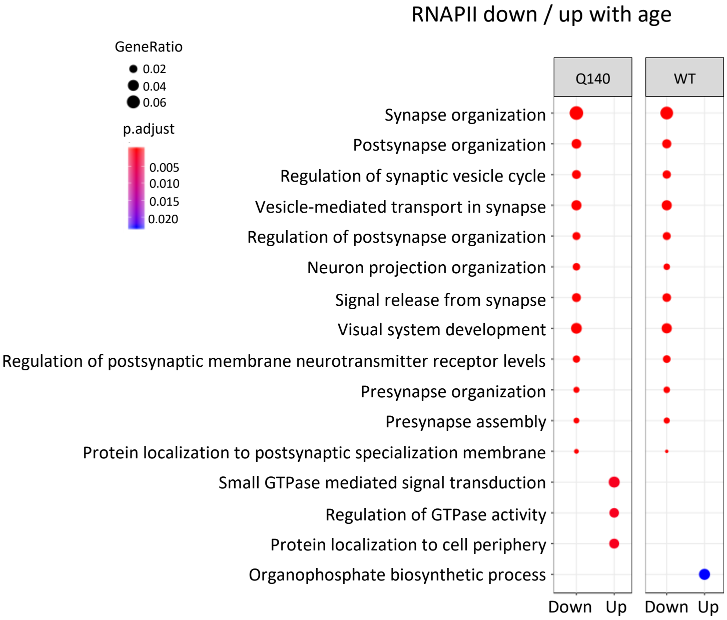

c

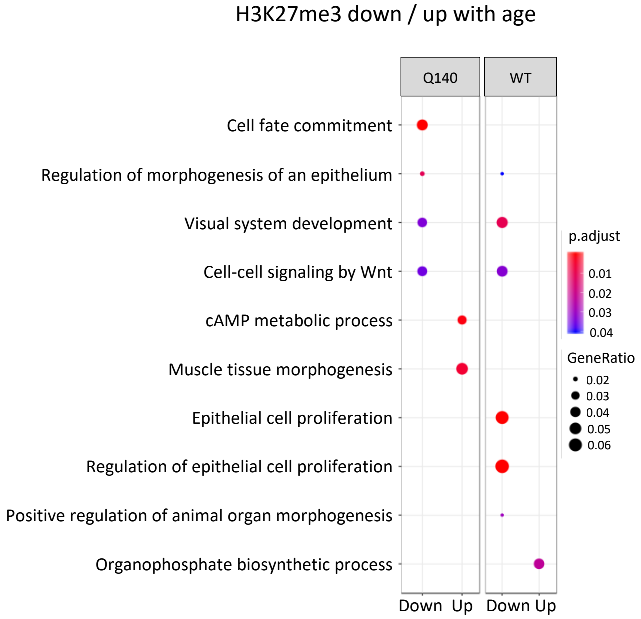

Supplementary Fig. 13

The effect of the HD mutation on age-related epigenetic changes in mouse striatum: complementary information and analyses

a, Summary table of regions showing differential enrichments in H3K27ac, H3K27me3 and RNAPII with age in Q140 or WT samples, processing male and female samples as replicates (adj. p value <0.05; Top). Summary table showing corresponding protein coding genes associated with differentially enriched regions in H3K27ac, H3K27me3 and RNAPII with age in Q140 and WT samples (Bottom). **b**, Gene Ontology analysis of regions differentially enriched in RNAPII in 6 vs 2 month striatal samples, in Q140 and WT contexts (FDR <0.05). Significant biological processes are shown using dot size proportional to gene ratio and heatmap reflecting adj. p value. **c**, Gene Ontology analysis of regions differentially enriched in H3K27me3 in 6 vs 2 month striatal samples, in Q140 and WT contexts (FDR <0.05). Significant biological processes are shown using dot size proportional to gene ratio and heatmap reflecting adj. p value. Source data are provided as a Source Data file.

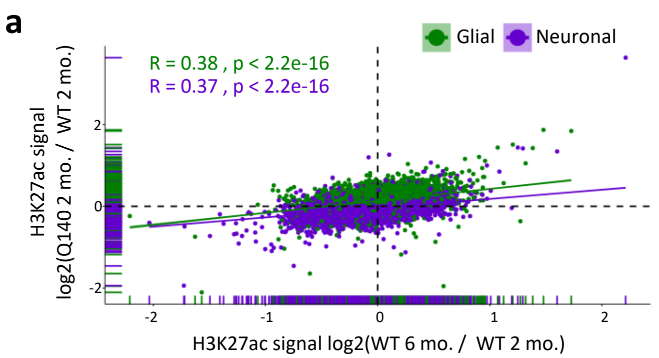

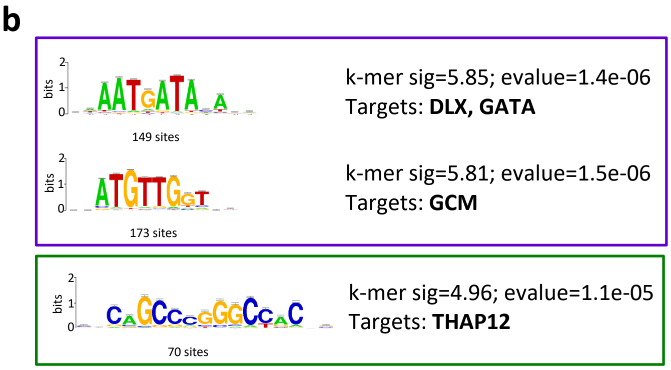

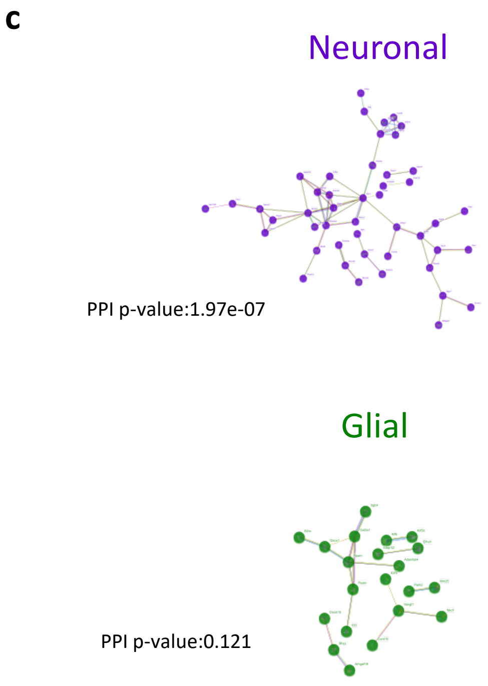

d

Term description	False Discovery Rate
**Biological process**	
Cell communication	0.0016
Signaling	0.0016
Regulation of signaling	0.0033
Neurogenesis	0.0043
Regulation of cell communication	0.0049
Nervous system development	0.0081
Cell surface receptor signaling pathway	0.0083
Chemical synaptic transmission	0.0083
Generation of neurons	0.0083
**Molecular function**	
Protein binding	0.00095
Signaling receptor binding	0.0053
Enzyme binding	0.0086
Protein phosphatase binding	0.0086
Neurotransmitter receptor activity	0.0086
Signaling receptor activity	0.0086
Molecular function regulator	0.0086
**Reactome Pathway**	
Protein-protein interactions at synapses	5.63E-06
Neuronal System	0.0012
Neurexins and neuroligins	0.0012
Class A/1 (Rhodopsin-like receptors)	0.0024
Unblocking of NMDA receptors, glutamate binding and activation	0.0036
Synaptic adhesion-like molecules	0.0047

Supplementary Fig. 14

The effect of the HD mutation on age-related epigenetic changes in mouse striatum: complementary information and analyses.

a, Scatter plot showing genotype-dependent variation in H3K27ac as a function of age-dependent variation in H3K27ac, at all neuronal- and glial-specific striatal enhancers. Log2 of fold change in H3K27ac levels between Q140 and WT samples at 2 months (log2(Q1402/WT2) are shown on y axis, log2 of fold change in H3K27ac levels between WT samples at 6 vs 2 months (log2(WT6/WT2) are shown on x axis. Spearman linear correlation was calculated for neuronal (purple) and glial (green) specific H3K27ac enriched regions, with correlation coefficient and p value shown for each cellular population. <0.05) shown in red. A binomial test was performed to assess enrichment in neurons or glia; * $P < 10^{-2}$. **b**, Results of DNA motif analysis, using differentially enriched H3K27ac regions both in Q140 vs WT samples at 2 months and in WT samples at 6 vs 2 months. Significantly enriched motifs are shown with corresponding statistics. **c**, STRING network representations of proteins encoded by genes close to differentially enriched H3K27ac regions ($p < 0.05$) both in Q140 vs WT samples at 2 months and in WT samples at 6 vs 2 months. On top, the protein network was built using proteins encoded by genes associated with neuronal-specific H3K27ac-enriched regions (neuronal-specific enhancers, purple), on the bottom, the protein network was built using proteins encoded by genes associated with glial-specific H3K27ac-enriched regions (glial-specific enhancers, green). Protein-protein Interaction p values reflecting network complexity are shown in each case. **d**, Summary table of STRING Gene Ontology network analysis using genes associated to neuronal-specific H3K27ac-enriched regions (neuronal-specific enhancers) showing H3K27ac depletion in both genotype- and age-dependent manners. GO terms (including Biological Process, Molecular Function and Reactome Pathway) with FDR < 0.01 are shown. Source data are provided as a Source Data file.

a

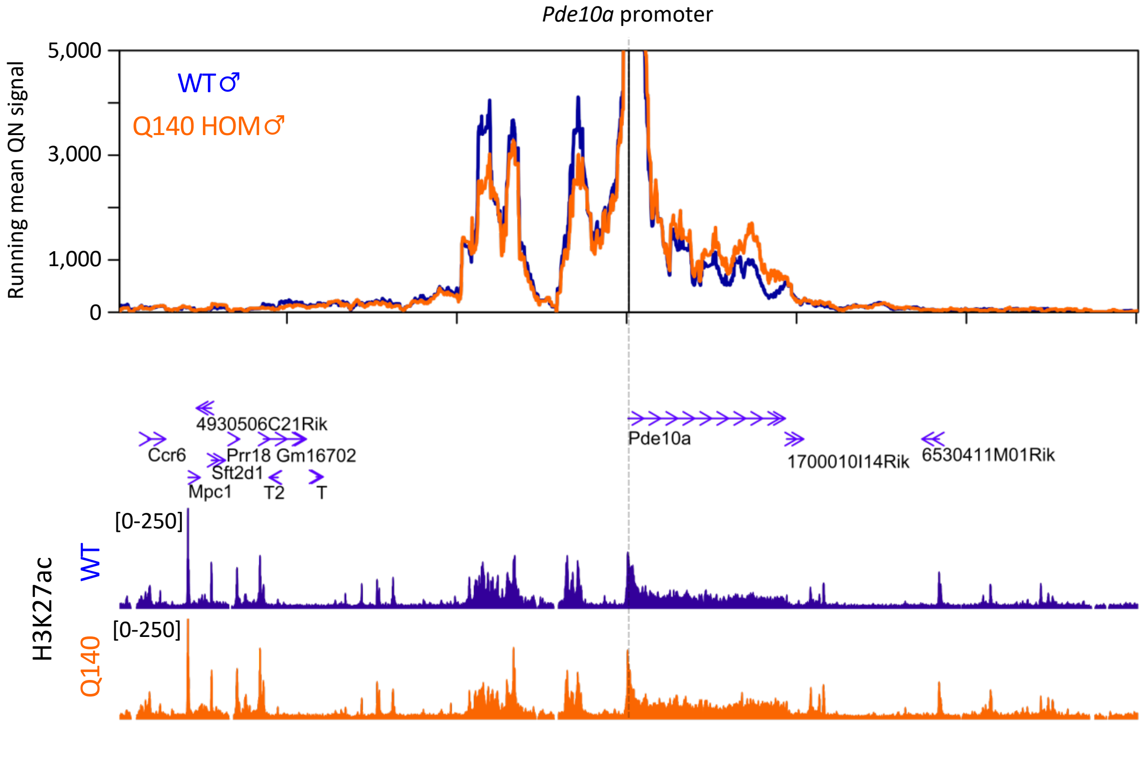

Chr17:8,202,775-9,402,364

b

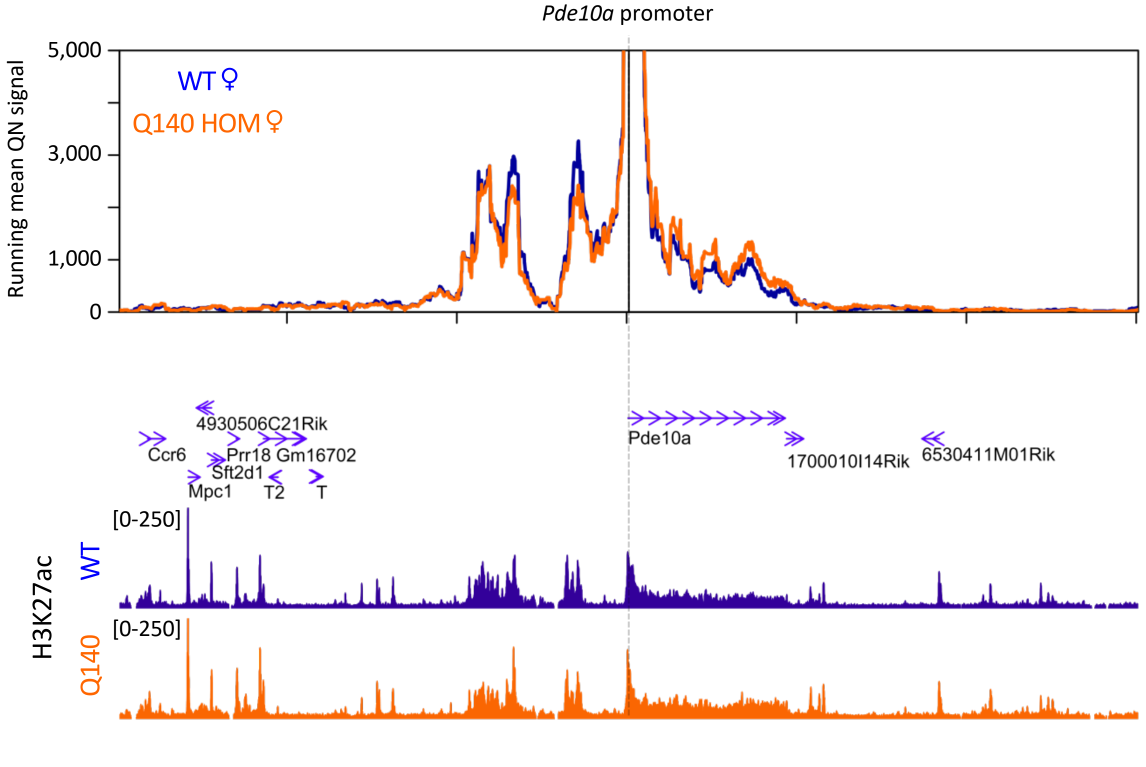

Chr17:8,202,775-9,402,364

Supplementary Fig. 15

3D chromatin architecture at *Pde10a* gene in the striatum of male and female HD Q140 mice

4C-seq profiles at *Pde10a* loci from Q140 (orange) and WT (blue) male (**a**) and female (**b**) mice striatum at 6 months of age. Gene annotations are included as well as H3K27ac ChIPseq signals (using ChIPseq data generated in this study on the striatum of Q140 and WT mice at same age).

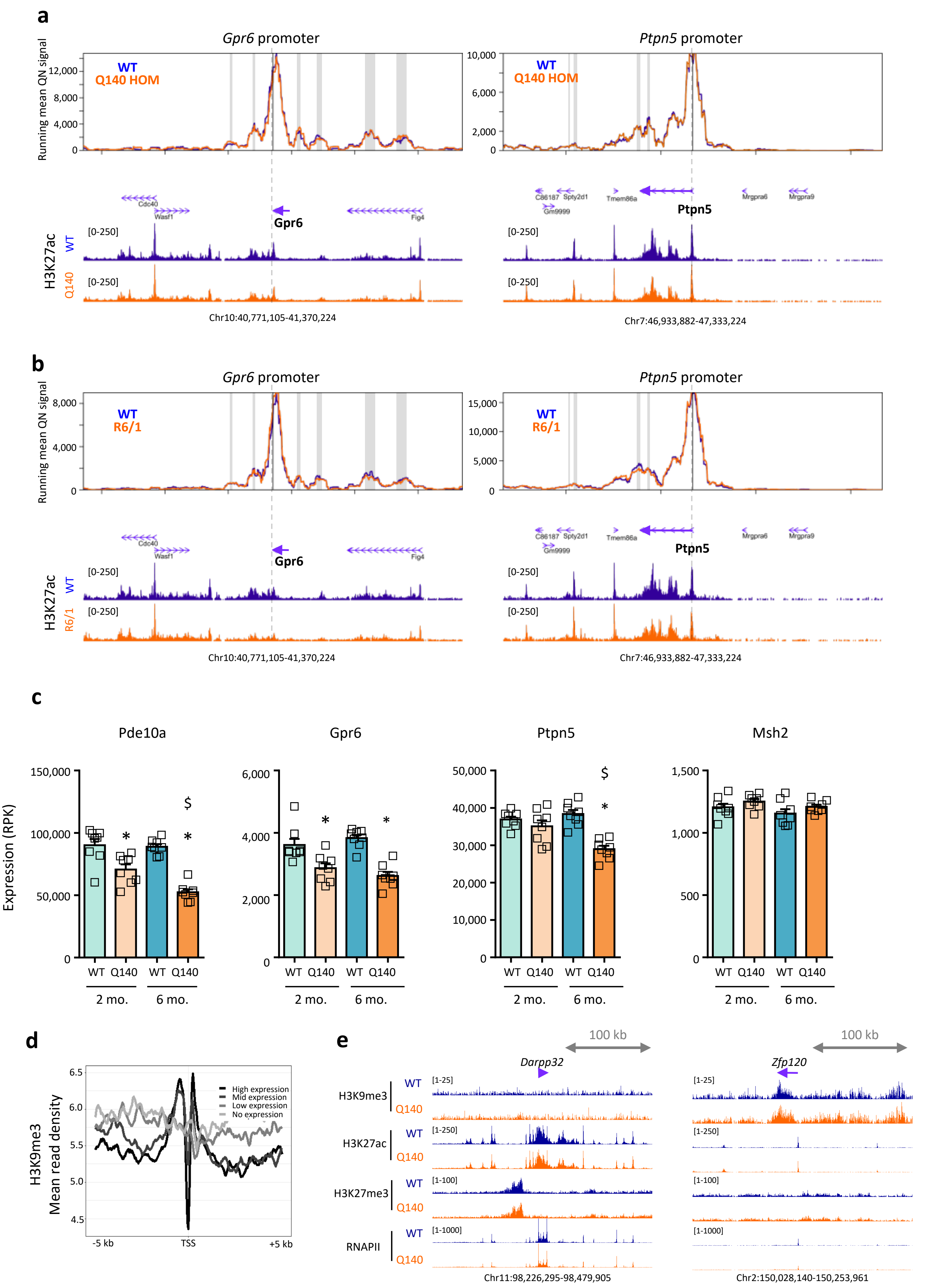
a
Gpr6 promoter
Ptpn5 promoter
Running mean QN signal
WT
Q140 HOM
12,000
8,000
4,000
0
10,000
6,000
2,000
Cdc40
Wasf1
Gpr6
Fig4
C86187
Spty2d1
Gm9999
Tmem86a
Ptpn5
Mrgpra6
Mrgpra9
H3K27ac
WT
Q140
[0-250]
Chr10:40,771,105-41,370,224
Chr7:46,933,882-47,333,224
b
Gpr6 promoter
Ptpn5 promoter
Running mean QN signal
WT
R6/1
8,000
4,000
0
15,000
10,000
5,000
H3K27ac
[0-250]
Chr10:40,771,105-41,370,224
Chr7:46,933,882-47,333,224
c
Pde10a
Gpr6
Ptpn5
Msh2
Expression (RPK)
150,000
100,000
50,000
0
6,000
4,000
2,000
50,000
40,000
30,000
20,000
10,000
1,500
1,000
500
WT Q140 WT Q140
2 mo. 6 mo.
*
$
d
H3K9me3
Mean read density
6.5
6.0
5.5
5.0
4.5
High expression
Mid expression
Low expression
No expression
-5 kb
TSS
+5 kb
e
100 kb
Darpp32
Zfp120
H3K9me3
H3K27ac
H3K27me3
RNAPII
WT
Q140
[1-25]
[1-250]
[1-100]
[1-1000]
Chr11:98,226,295-98,479,905
Chr2:150,028,140-150,253,961

Supplementary Fig. 16

3D chromatin architecture at super-enhancer regulated genes in the striatum of HD Q140 mice: complementary information and analyses

a, 4C-seq profiles at *Gpr6* and *Ptpn5* loci from Q140 (orange) and WT (blue) striatum at 6 months of age. The mean of male and female 4C-seq quartile normalized read counts is plotted as the main lane for each condition. Gene annotations are included as well as H3K27ac ChIPseq signals (using ChIPseq data generated in this study on the striatum of Q140 and WT mice at same age). Grey shadows show specific interacting regions. **b**, 4C-seq profiles at *Gpr6* and *Ptpn5* loci from R6/1 (orange) and WT (blue) striatum at 14 weeks of age. Gene annotations and H3K27ac ChIPseq signals [3] are included. Grey shadow show specific interacting regions. **c**, Bar graphs showing expression of *Pde10a*, *Gpr6*, *Ptpn5* and *Msh2* in WT and Q140 striatum at 2 and 6 months of age. Expression values were computed from RNAseq data generated in Q140 mice[5], using DESeq2 Bioconductor package. N= 8 biological replicates per group. RPK, reads per kilobases. The Benjamini and Hochberg method was used for multiple testing correction. Pde10a: *, adj. *p* val = $4x10^{-2}$ Q140 vs WT at 2 months; *, adj. *p* val = $2x10^{-13}$ Q140 vs WT at 6 months; $, adj. *p* val =$3x10^{-5}$ 6 vs 2 months in Q140. Gpr6: *, adj. *p* val = $4x10^{-2}$ Q140 vs WT at 2 months; *, adj. *p* val = $2x10^{-7}$ Q140 vs WT at 6 months; Ptpn5: *, adj. *p* val = $9x10^{-8}$ Q140 vs WT at 6 months; $, adj. *p* val =$2x10^{-4}$ 6 vs 2 months in Q140s **d**, Representative gene metaprofiles for H3K9me3 at no, low-, mid- and high-expressed genes in the striatum. No-, low-, mid- and high-expressed genes were delineated according to quartile distribution of normalized striatal RNAseq values (i.e. no-expressed < 1st, 1st <low-expressed < 2nd, 2nd<mid-expressed<3rd, high-expressed>4th quartiles). H3K9me3 ChIPseq data and RNAseq data generated on WT striatum at 6 months were used in the analysis. TSS, Transcription Start Site. **e,** UCSC genome browser capture showing representative H3K9me3, H3K27ac, H3K27me3 and RNAPII signal in the striatum of WT and Q140 mouse striatum at 6 months at selected loci, including euchromatinized region (*Darpp32* locus) and heterochromatinized region (*Zfp120* gene in chromosome 2*).* Source data are provided as a Source Data file.

Figure S17

a

Pearson Correlation coefficients

	WT ♀	WT ♂	Q140 ♀	Q140 ♂
**WT ♀**	1	0.873	0.86	0.804
**WT ♂**	0.873	1	0.821	0.856
**Q140 ♀**	0.86	0.821	1	0.804
**Q140 ♂**	0.804	0.856	0.804	1

b

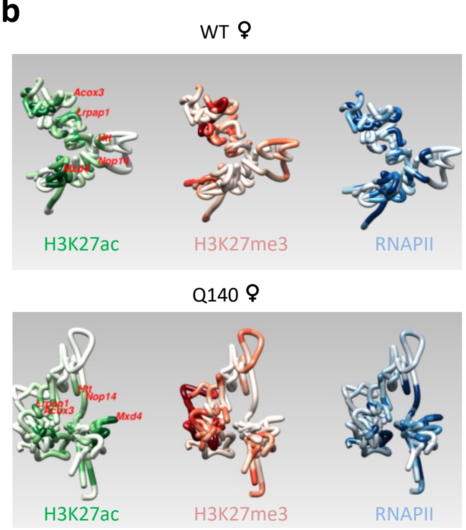

c

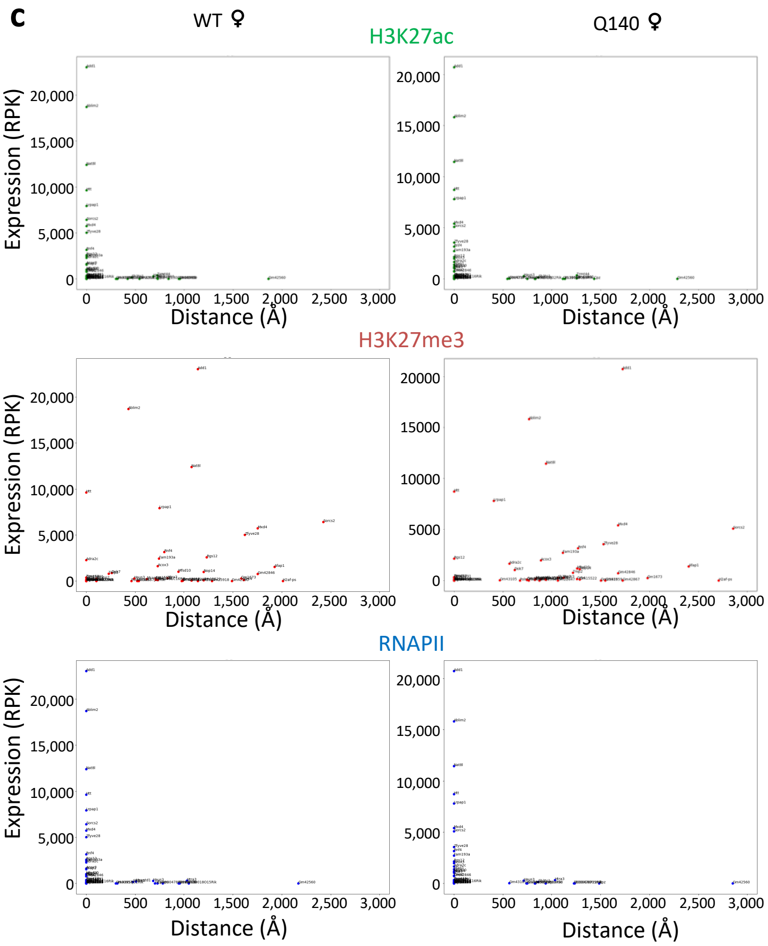

d

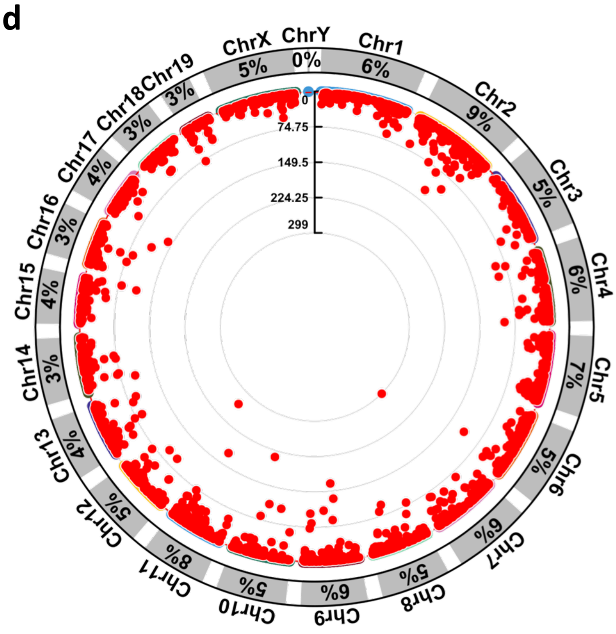

f

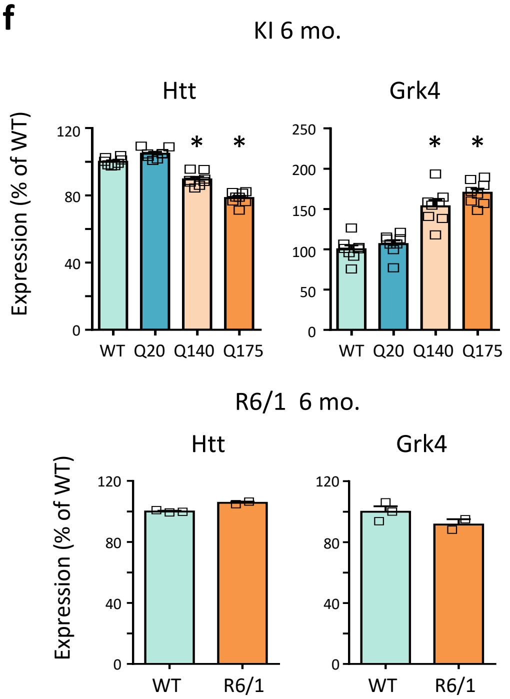

e

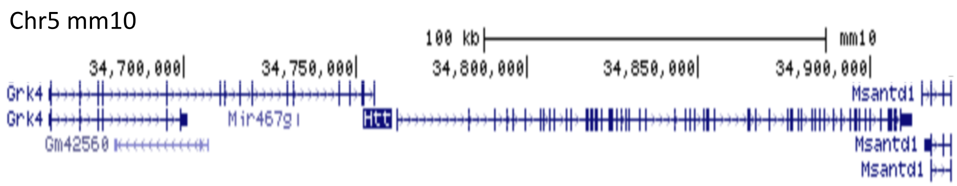

Supplementary Fig. 17

The HD mutation induces disease locus-specific alterations of 3D chromatin architecture and transcription regulation in the striatum of Q140 mice

a, Summary table of Pearson's correlation coefficients, comparing the different vHi-C models encompassing *Htt*, and generated using 4Cseq data on WT and Q140 samples (N=2, 1 ♂ + 1 ♀). **b**, Representative 3D models at the region encompassing *Htt*, in WT and Q140 striatum. Chromatin painting was performed using H3K27ac (green), H3K27me3 (red) and RNAPII (blue) ChIP-seq data on the striatum of WT or Q140 mice at 6 months. Viewpoints are depicted with a red label and names are indicated to improve visualization of 3D chromatin architecture. **c,** Dot plots showing expression of genes located in the two-megabase region reconstructed by vHi-C according to their spatial proximity (Armstrongs, Å) to previously identified peak in H3K27ac, H3K27me3 and RNAPII ChIP-seq data generated on WT and Q140 striatum (using ♀ data). **d**, Manhattan plot representing the distribution of differentially expressed genes (DEG) in R6/1 vs WT striatum at 6 months across the different chromosomes. Significant DEG (adj. p value <0.01) are labelled as red dots and the percentage of DEG within each chromosome is shown in peripheral arc. RNAseq data were analysed using R6/1 transcriptomic database and DESeq2 Bioconductor package [3] **e,** UCSC genome browser capture showing *Grk4* and *Htt* genes. **f,** Bar graphs showing mRNA levels of *Htt* and *Grk4* in the striatum of 6 month-old HD KI mice expressing *Htt* with 175 CAG (Q175), 140 CAG (Q140), 20 CAG (Q20) and in the striatum of WT mice. RNAseq data were analysed using transcriptomic database and DESeq2 Bioconductor package [5]. N= 8 biological replicates per group. Expression levels are represented as percentage of WT values. The Benjamini and Hochberg method was used for multiple testing correction. Htt: *, adj. *p* val = 6×10^{-7} Q140 vs WT ; *, adj. *p* val = 10^{-33} Q175 vs WT. Grk4: *, adj. *p* val = 10^{-7} Q140 vs WT ; *, adj. *p* val = 10^{-12} Q175 vs WT (top). Bargraphs showing mRNA levels of *Htt* and *Grk4* in the striatum of 6 month-old R6/1 and WT mice. RNAseq data were analysed using R6/1 transcriptomic database(GSE59572; https://www.ncbi.nlm.nih.gov/geo/query/acc.cgi?acc=GSE59572) and DESeq2 Bioconductor package [3]. N= 3 and 2 biological replicates for WT and R6/1 samples, respectively. Expression levels are represented as percentage of WT values. The Benjamini and Hochberg method was used for multiple testing correction (bottom). Source data are provided as a Source Data file.

Pde10a 4C-seq reading	CGTCAGGAATCCAGCGGTTTAGATC
Pde10a 4C-seq non-reading	GTGCGCACCGAGCCTTTG
Gpr6 4C-seq reading	CCAGCAAGAAGGGCAGAGATC
Gpr6 4C-seq non-reading	GCACGGGTTTTATTTTCAGCCC
Htt 4C-seq reading	GGCTTCGGGGTCCACG
Htt 4C-seq non-reading	CCGGGCATGGTGGTGCA
Dclk3 4C-seq reading	GGCAGGAGACTCAGCATCTGATC
Dclk3 4C-seq non-reading	GCCCACAGATTTTTGCAGAGG
Ptpn5 4C-seq reading	TCCTGGTGCGCTTTTCAAAAGGATC
Ptpn5 4C-seq non-reading	CTTCCCTTTCTCCCGTTACCTCTT
Msh2 4C-seq reading	GTGGGGGAGAGGTTATTATGGTGATC
Msh2 4C-seq non-reading	TGGTTGACCTGGAACTCAGTAGGTTT
Acox3 4C-seq reading	GCTCTGTGGGCTTTGTTTGTTTGATC
Acox3 4C-seq non-reading	GCCCTAGTTTTGGTCTGAAGTCCTAT
Mxd4 4C-seq reading	TCCTGGCTGCAGTGGGATC
Mxd4 4C-seq non-reading	GTGCAAGGGAGGGAGGAGTC
Nop14 4C-seq reading	GCCTTAAACTAGCCAGCAAAACGATC
Nop14 4C-seq non-reading	CAGCACCCCTCATGACTTCTACA
Lrpap1 4C-seq reading	TGGGGTGGGCGGGATC
Lrpap1 4C-seq non-reading	CCTAGTCTCTGCACCTGCAGC

Supplementary Table 1
List of primers used for 4Cseq experiments
