# Supplementary material for: Age-related and disease locus-specific mechanisms contribute to early remodelling of chromatin structure in Huntington’s disease mice
Source: Nat Commun. 2021 Jan 13;12:364. doi: 10.1038/s41467-020-20605-2 (PMC7807045; doi:10.1038/s41467-020-20605-2)
Supplement: Supplementary file 3 — Description of Additional Supplementary Files [file 41467_2020_20605_MOESM3_ESM.pdf]

Description of Additional Supplementary Files

Supplementary Data 1: Regions identified as neuronal-, glial- and non-specific from mouse striatal ChIPseq data

Supplementary Data 2: Interacting regions identified in 4Cseq data experiments in WT and Q140 striatum

Supplementary Movie 1: Three-dimensional model reconstruction of Htt vHi-C data using WT mouse striatum (using female sample) and superposing with H3K27ac, H3K27me3 and RNAPII signals of striatal ChIPseq data

Supplementary Movie 2: Three-dimensional model reconstruction of Htt vHi-C data using Q140 mouse striatum (using female sample) and superposing with H3K27ac, H3K27me3 and RNAPII signals of striatal ChIPseq data
