# Supplementary material for: Age-related and disease locus-specific mechanisms contribute to early remodelling of chromatin structure in Huntington’s disease mice
Source: Nat Commun. 2021 Jan 13;12:364. doi: 10.1038/s41467-020-20605-2 (PMC7807045; doi:10.1038/s41467-020-20605-2)
Supplement: Supplementary file 8 — Reporting Summary [file 41467_2020_20605_MOESM8_ESM.pdf]

nature research

Corresponding author(s): Merienne

Last updated by author(s): 2020/10/26

Reporting Summary

Nature Research wishes to improve the reproducibility of the work that we publish. This form provides structure for consistency and transparency in reporting. For further information on Nature Research policies, see Authors & Referees and the Editorial Policy Checklist .

Statistics

For all statistical analyses, confirm that the following items are present in the figure legend, table legend, main text, or Methods section.

n/a	Confirmed	
☐	☒	The exact sample size (*n*) for each experimental group/condition, given as a discrete number and unit of measurement
☐	☒	A statement on whether measurements were taken from distinct samples or whether the same sample was measured repeatedly
☐	☒	The statistical test(s) used AND whether they are one- or two-sided<br>*Only common tests should be described solely by name; describe more complex techniques in the Methods section.*
☒	☐	A description of all covariates tested
☐	☒	A description of any assumptions or corrections, such as tests of normality and adjustment for multiple comparisons
☐	☒	A full description of the statistical parameters including central tendency (e.g. means) or other basic estimates (e.g. regression coefficient) AND variation (e.g. standard deviation) or associated estimates of uncertainty (e.g. confidence intervals)
☐	☒	For null hypothesis testing, the test statistic (e.g. *F*, *t*, *r*) with confidence intervals, effect sizes, degrees of freedom and *P* value noted<br>*Give P values as exact values whenever suitable.*
☒	☐	For Bayesian analysis, information on the choice of priors and Markov chain Monte Carlo settings
☒	☐	For hierarchical and complex designs, identification of the appropriate level for tests and full reporting of outcomes
☐	☒	Estimates of effect sizes (e.g. Cohen's *d*, Pearson's *r*), indicating how they were calculated

Our web collection on statistics for biologists contains articles on many of the points above.

Software and code

Policy information about availability of computer code

Data collection	Equipment software of FACS Aria Fusion and FACS Aria II BD FACSDiva v8 and Illumina Hiseq 4000
Data analysis	Sabre, Bowtie (v.1.0.0.), Sicer (v1.1.), Homer Annotate Peaks (v4.9.1), SeqMiner (1.3.4), RSAT peak-motifs, 4cin, 4see, DESeq2(v.1.14), Tophat2, HTSeq (v0.6.1p1), Microsoft Excel 2010, R (v.3.3.2), fastqc (v.0.11.5.), RTA (v.2.7.3), bcl2fastq (v.2.17.1.14.), Snapgene (v1.1.3), STRING (v.11.0), peakC, UCSC genome browser, Clustvis 2.0, NDP.view v2, Icy , GREAT (v4.0.4), BEDTools (v2.28), Rstudio (v.1.1.456) and R packages: CMplot, ggpubR, Bioconductor, Clusterprofiler.

For manuscripts utilizing custom algorithms or software that are central to the research but not yet described in published literature, software must be made available to editors/reviewers. We strongly encourage code deposition in a community repository (e.g. GitHub). See the Nature Research guidelines for submitting code & software for further information.

Data

Policy information about availability of data

All manuscripts must include a data availability statement. This statement should provide the following information, where applicable:

- Accession codes, unique identifiers, or web links for publicly available datasets
- A list of figures that have associated raw data
- A description of any restrictions on data availability

ChIPseq and 4Cseq have been deposited at Gene Expression Omnibus repository (GSE144684 [https://www.ncbi.nlm.nih.gov/geo/query/acc.cgi?acc=GSE144684] and GSE144699 [https://www.ncbi.nlm.nih.gov/geo/query/acc.cgi?acc=GSE144699], respectively)

Field-specific reporting

Please select the one below that is the best fit for your research. If you are not sure, read the appropriate sections before making your selection.

- [x] Life sciences
- [ ] Behavioural & social sciences
- [ ] Ecological, evolutionary & environmental sciences

For a reference copy of the document with all sections, see nature.com/documents/nr-reporting-summary-flat.pdf

Life sciences study design

All studies must disclose on these points even when the disclosure is negative.

Sample size	No statistical methods were used to predetermine sample sizes in ChIPseq and 4Cseq experiments, but our sample sizes were similar to those reported in similar studies (Vashishtha et al. 2013; Achour et al. 2015; Chatterjee et al. 2018). For behavioral analyses, sample sizes were chosen based on previous studies using similar experimental designs (Stefanko et al. 2017; Galvan et al. 2018).
Data exclusions	No data were excluded from the analysis
Replication	All ChIPseq and 4Cseq data were replicated. PCA, correlative heatmaps and scatter plot analyses were used to assess data reproducibility. All attempts at replication were successful. Behavioral experiments were not replicated.
Randomization	For ChIPseq and 4Cseq experiments using pooled mouse striatal tissues, male and female tissues were processed independently. One replicate was generated using males tissues, and the other replicate using female samples. For behavioral analyses, HD and WT animals were randomized.
Blinding	Blinding could not be applied to epigenomic studies, because samples had to be controlled by condition and the individual implicated in the production of next generation sequencing data was also implicated in data analysis. Behavioral experiments were performed blind by two experimentators. FANS and immunostaining analyses were conducted blindly to experimentor, using an automated software analysis with identical parameters along the different biological replicates and groups.

Reporting for specific materials, systems and methods

We require information from authors about some types of materials, experimental systems and methods used in many studies. Here, indicate whether each material, system or method listed is relevant to your study. If you are not sure if a list item applies to your research, read the appropriate section before selecting a response.

Materials & experimental systems

n/a	Involved in the study	
[ ]	[x]	Antibodies
[x]	[ ]	Eukaryotic cell lines
[x]	[ ]	Palaeontology
[ ]	[x]	Animals and other organisms
[x]	[ ]	Human research participants
[x]	[ ]	Clinical data

Methods

n/a	Involved in the study	
[ ]	[x]	ChIP-seq
[ ]	[x]	Flow cytometry
[x]	[ ]	MRI-based neuroimaging

Antibodies

Antibodies used

Rabbit polyclonal anti-Histone H3 (acetyl K27) - ChIP Grade, Abcam, Cat# ab4729, RRID:AB_2118291
H3K27me3 rabbit polyclonal antibody ChIP-seq grade, Diagenode, Cat# C15410195, RRID:AB_2753161
7C2 RNA Polymerase II antibody Besse et al. 1995 (Home made, IGBMC Strasbourg)
Anti-Histone H3 (try methyl K9) antibody - ChIP Grade, Abcam, Cat# ab8898, RRID:AB_306848
Anti-NeuN, clone A60 mouse monoclonal antibody, Millipore, Cat# MAB377, RRID:AB_2298772
Donkey anti-Mouse IgG (H+L) Highly Cross-Adsorbed Secondary Antibody, Alexa Fluor 488, Invitrogen, Cat# A-21202, RRID:AB_141607
Donkey anti-Rabbit IgG (H+L) Highly Cross-Adsorbed Secondary Antibody, Alexa Fluor 594, Invitrogen, Cat# A-21207, RRID:AB_141637
Sox9 (D8G8H) Rabbit mAb #82630, Cell Signaling, Cat# 82630S, RRID:AB_2665492
RBFOX3/NeuN Antibody (1B7) [Alexa Fluor® 405], Novus Biologicals, Cat# NBP1-92693AF405

Validation	H3K27ac, H3K27me3, H3K9me3 and RNAPII antibodies used in ChIPseq experiments are ChIPseq grade and were used in mice for same application in previous studies (Fukuda et al. 2014; Achour et al., 2015; von Schimmelmann et al. 2016; Palmisano et al. 2019).<br>The NeuN antibody was used in FANS-based experiments using mouse brain tissues in previous studies (Halder et al. 2015; von Schimmelmann et al. 2016; Fernandez-Albert et al. 2019)

Animals and other organisms

Policy information about studies involving animals; ARRIVE guidelines recommended for reporting animal research

Laboratory animals	HD Q140 knockin mice (140ChdiJ), C57BL/6J, males and females, 2 and 6 months of age.<br>HD R6/1 mice (6 N/A B6.Cg-Tg(HDexon1)61Gpb/J), C57BL/6J, males, 3 months of age.<br>Mice were housed in a controlled-temperature room maintained on a 12h light/dark cycle. Food and water were available ad libidum.
Wild animals	The study did not involve wild animals.
Field-collected samples	The study did not involve samples collected from the field.
Ethics oversight	All animal procedures were approved by local ethics committee (CREMEAS) and French Research Ministry (no. APAFIS#4301-2016022912385206v2 and no. APAFIS#504-2015042011568820_v3)

Note that full information on the approval of the study protocol must also be provided in the manuscript.

ChIP-seq

Data deposition

- [x] Confirm that both raw and final processed data have been deposited in a public database such as GEO.
- [x] Confirm that you have deposited or provided access to graph files (e.g. BED files) for the called peaks.

Data access links<br>*May remain private before publication.*	ChIPseq and 4Cseq data have been deposited at Gene Expression Omnibus repository (GSE144684 [https://www.ncbi.nlm.nih.gov/geo/query/acc.cgi?acc=GSE144684] and GSE144699 [https://www.ncbi.nlm.nih.gov/geo/query/acc.cgi?acc=GSE144699], respectively)
Files in database submission	input_WT_6m_M_rep3.fastq.gz<br>input_Q140_6m_M_rep3.fastq.gz<br>H3K9me3_WT_6m_M_rep3.fastq.gz<br>H3K27me3_WT_6m_M_rep3.fastq.gz<br>H3K9me3_Q140_6m_M_rep3.fastq.gz<br>H3K27me3_Q140_6m_M_rep3.fastq.gz<br>input_WT_6m_F_rep4.fastq.gz<br>input_Q140_6m_F_rep4.fastq.g<br>H3K27ac_WT_6m_F_rep4.fastq.gz<br>H3K27me3_WT_6m_F_rep4.fastq.gz<br>RNAPII_WT_6m_F_rep4.fastq.gz<br>H3K27ac_Q140_6m_F_rep4.fastq.gz<br>H3K27me3_Q140_6m_F_rep4.fastq.gz<br>RNAPII_Q140_6m_F_rep4.fastq.gz<br>input_WT_2m_F_rep2.fastq.gz<br>input_Q140_2m_F_rep2.fastq.gz<br>H3K27ac_WT_2m_F_rep2.fastq.gz<br>H3K27me3_WT_2m_F_rep2.fastq.gz<br>RNAPII_WT_2m_F_rep2.fastq.gz<br>H3K27ac_Q140_2m_F_rep2.fastq.gz<br>H3K27me3_Q140_2m_F_rep2.fastq.gz<br>RNAPII_Q140_2m_F_rep2.fastq.gz<br>input_WT_20w_M_rep1_NeuN+.fastq.gz<br>input_WT_20w_M_rep1_NeuN-.fastq.gz<br>H3K27ac_WT_20w_M_rep1_NeuN+.fastq.gz<br>H3K27me3_WT_20w_M_rep1_NeuN+.fastq.gz<br>H3K27ac_WT_20w_M_rep1_NeuN-.fastq.gz<br>H3K27me3_WT_20w_M_rep1_NeuN-.fastq.gz<br>input_WT_2m_M_rep3.fastq.gz<br>input_Q140_2m_M_rep3.fastq.gz<br>H3K27ac_WT_2m_M_rep3.fastq.gz<br>H3K27me3_WT_2m_M_rep3.fastq.gz<br>RNAPII_WT_2m_M_rep3.fastq.gz

H3K27ac_Q140_2m_M_rep3.fastq.gz
H3K27me3_Q140_2m_M_rep3.fastq.gz
RNAPII_Q140_2m_M_rep3.fastq.gz
H3K27me3_WT_20w_M_rep2_NeuN+.fastq.gz
H3K27me3_WT_20w_M_rep2_NeuN-.fastq.gz
input_WT_20w_M_rep2_NeuN+.fastq.gz
input_WT_20w_M_rep2_NeuN-.fastq.gz
H3K27ac_WT_20w_M_rep2_NeuN+.fastq.gz
H3K27ac_WT_20w_M_rep2_NeuN-.fastq.gz
input_WT_6m_M_rep3.wig.gz
input_Q140_6m_M_rep3.wig.gz
H3K9me3_WT_6m_M_rep3.wig.gz
H3K27me3_WT_6m_M_rep3.wig.gz
H3K9me3_Q140_6m_M_rep3.wig.gz
H3K27me3_Q140_6m_M_rep3.wig.gz
input_WT_6m_F_rep4.wig.gz
input_Q140_6m_F_rep4.wig.gz
H3K27ac_WT_6m_F_rep4.wig.gz
H3K27me3_WT_6m_F_rep4.wig.gz
RNAPII_WT_6m_F_rep4.wig.gz
H3K27ac_Q140_6m_F_rep4.wig.gz
H3K27me3_Q140_6m_F_rep4.wig.gz
RNAPII_Q140_6m_F_rep4.wig.gz
input_WT_2m_F_rep2.wig.gz
input_Q140_2m_F_rep2.wig.gz
H3K27ac_WT_2m_F_rep2.wig.gz
H3K27me3_WT_2m_F_rep2.wig.gz
RNAPII_WT_2m_F_rep2.wig.gz
H3K27ac_Q140_2m_F_rep2.wig.gz
H3K27me3_Q140_2m_F_rep2.wig.gz
RNAPII_Q140_2m_F_rep2.wig.gz
input_WT_20w_M_rep1_NeuN+.wig.gz
input_WT_20w_M_rep1_NeuN-.wig.gz
H3K27ac_WT_20w_M_rep1_NeuN+.wig.gz
H3K27me3_WT_20w_M_rep1_NeuN+.wig.gz
H3K27ac_WT_20w_M_rep1_NeuN-.wig.gz
H3K27me3_WT_20w_M_rep1_NeuN-.wig.gz
input_WT_2m_M_rep3.wig.gz
input_Q140_2m_M_rep3.wig.gz
H3K27ac_WT_2m_M_rep3.wig.gz
H3K27me3_WT_2m_M_rep3.wig.gz
RNAPII_WT_2m_M_rep3.wig.gz
H3K27ac_Q140_2m_M_rep3.wig.gz
H3K27me3_Q140_2m_M_rep3.wig.gz
RNAPII_Q140_2m_M_rep3.wig.gz
H3K27me3_WT_20w_M_rep2_NeuN+.wig.gz
H3K27me3_WT_20w_M_rep2_NeuN-.wig.gz
input_WT_20w_M_rep2_NeuN+.wig.gz
input_WT_20w_M_rep2_NeuN-.wig.gz
H3K27ac_WT_20w_M_rep2_NeuN+.wig.gz
H3K27ac_WT_20w_M_rep2_NeuN-.wig.gz

Genome browser session
(e.g. UCSC)

http://genome.ucsc.edu/s/rafalcala/Q140HD_mm10

Methodology

Replicates

We have generated a minimum of two independent biological replicates fro ChIP-seq experiments, with exception of H3K9me3, for which only one biological replicate was generated for preliminar assessment

Sequencing depth

ChIP-seq samples were sequenced in Illumina Hiseq4000 in a pair-end 50 bp length configuration with exception of the second replicate for NeuN sorted based experiments, for which 100 bp length configuration was used. All samples showed >90% reads with high quality mapping to mm10

Antibodies

Rabbit polyclonal anti-Histone H3 (acetyl K27) - ChIP Grade Abcam Cat# ab4729, RRID:AB_2118291
H3K27me3 rabbit polyclonal antibody ChIP-seq grade Diagenode Cat# C15410195, RRID:AB_2753161
7C2 RNA Polymerase II antibody Besse et al. 1995 (Home made, IGBMC Strasbourg)
Anti-Histone H3 (try methyl K9) antibody - ChIP Grade Abcam Cat# ab8898, RRID:AB_306848

Peak calling parameters	Sequence reads were mapped to reference genome mm10 using Bowtie 1.0.0 with the following parameters -m 1 --strata --best -y -S -l 40 -p 2. Peak detection of H3K27ac, RNAPII and H3K27me3 was performed using SICER59,60 v1.1 with the following parameters: window size: 200; e-value: 0.003. Gap size parameters were selected according to the score value estimated by statistical method implemented in SICER: selected values of gap size are 1000, 600 and 1400 for H3K27ac, RNAPII and H3K27me3, respectively
Data quality	Only peaks with and FDR < 0.05 were used for initial peak calling and differences among conditions were considered only for FDR < 10-5
Software	fastqc (v.0.11.5.), RTA (v.2.7.3), bcl2fastq (v.2.17.1.14.), Bowtie (v.1.0.0.), Bedtools (v.2.22.0), Sicer (v1.1.), Homer Annotate Peaks (v4.9.1), SeqMiner (1.3.4), RSAT peak-motifs, DESeq2(v.1.14)

Flow Cytometry

Plots

Confirm that:

- [x] The axis labels state the marker and fluorochrome used (e.g. CD4-FITC).
- [x] The axis scales are clearly visible. Include numbers along axes only for bottom left plot of group (a 'group' is an analysis of identical markers).
- [x] All plots are contour plots with outliers or pseudocolor plots.
- [x] A numerical value for number of cells or percentage (with statistics) is provided.

Methodology

Sample preparation	Mice were killed by cervical dislocation, striata were microdissected and fast-freeze on liquid nitrogen. Nuclei were extracted after cross-linking (ChIP-seq) or in native conditions (NeuN population analysis) by mechanical tissue disruption and low detergent based nuclear isolation procedure. Nuclei were stained with a-NeuN antibody (Millipore Cat# MAB377) and Alexa-Fluor 488 (ThermoFisher Scientific Cat# A-21202) secondary antibody for ChIP-seq experiments and with RBFOX3/NeuN Antibody (1B7) [Alexa Fluor® 405] for NeuN population analysis
Instrument	FACS ARIA FUSION and FACS ARIA II
Software	BD FACSDiva v8
Cell population abundance	The purity of post-sorted fraction were >90-95% as determined by post-sorting analysis of purified fractions
Gating strategy	Nuclei were first separated from remanent debri according to their size and granularity (FSC-A vs SSC-A). Singlets were gated in the linear relation between FSC-A and FSC-H. Finally, NeuN + and NeuN- nuclei were separated by their fluorescent signal of AF488 (ChIP-seq experiments) or AF405 (NeuN population analysis). Population boundaries were clearly separated and post-sorting controls were performed.

- [x] Tick this box to confirm that a figure exemplifying the gating strategy is provided in the Supplementary Information.
